# Comparative Genomics Reveals High Genomic Diversity in the Genus *Photobacterium*

**DOI:** 10.3389/fmicb.2017.01204

**Published:** 2017-06-29

**Authors:** Henrique Machado, Lone Gram

**Affiliations:** ^1^Department of Biotechnology and Biomedicine, Technical University of Denmark, MatematiktorvetKgs Lyngby, Denmark; ^2^Novo Nordisk Foundation Center for Biosustainability, Technical University of DenmarkHørsholm, Denmark

**Keywords:** *Photobacterium*, *Vibrionaceae*, comparative genomics, pan-genome, core-genome

## Abstract

*Vibrionaceae* is a large marine bacterial family, which can constitute up to 50% of the prokaryotic population in marine waters. *Photobacterium* is the second largest genus in the family and we used comparative genomics on 35 strains representing 16 of the 28 species described so far, to understand the genomic diversity present in the *Photobacterium* genus. Such understanding is important for ecophysiology studies of the genus. We used whole genome sequences to evaluate phylogenetic relationships using several analyses (16S rRNA, MLSA, *fur*, amino-acid usage, ANI), which allowed us to identify two misidentified strains. Genome analyses also revealed occurrence of higher and lower GC content clades, correlating with phylogenetic clusters. Pan- and core-genome analysis revealed the conservation of 25% of the genome throughout the genus, with a large and open pan-genome. The major source of genomic diversity could be traced to the smaller chromosome and plasmids. Several of the physiological traits studied in the genus did not correlate with phylogenetic data. Since horizontal gene transfer (HGT) is often suggested as a source of genetic diversity and a potential driver of genomic evolution in bacterial species, we looked into evidence of such in *Photobacterium* genomes. Genomic islands were the source of genomic differences between strains of the same species. Also, we found transposase genes and CRISPR arrays that suggest multiple encounters with foreign DNA. Presence of genomic exchange traits was widespread and abundant in the genus, suggesting a role in genomic evolution. The high genetic variability and indications of genetic exchange make it difficult to elucidate genome evolutionary paths and raise the awareness of the roles of foreign DNA in the genomic evolution of environmental organisms.

## Introduction

Oceans cover 70% of Planet Earth and it has been estimated to harbor an extensive unexplored genomic potential (Sunagawa et al., 2015). The rapid development in sequencing technologies and the focus on improved cultivation of hitherto uncultured microorganisms has dramatically increased our understanding of both diversity and biotechnological potential found in the marine habitats (Zengler et al., 2002; Lasken, 2012; Mardis, 2013; Choi et al., 2015; Loman and Pallen, 2015; Sunagawa et al., 2015). *Vibrionaceae* is a prominent marine bacterial family and it represents 0.8% of the bacterial population as found in the *Tara* oceans metagenomic data (Sunagawa et al., 2015). Based on molecular assessment, it can constitute up to 50% of the prokaryotic population in marine waters (Wietz et al., 2010a; Gilbert et al., 2012). *Vibrio* is the largest genus within this family (73% in *Tara* oceans data) and it has been extensively studied, in part due to the importance of the human pathogen *V. cholerae* (Meibom et al., 2004; Stauder et al., 2012; Kim et al., 2015; Papenfort et al., 2015; Rajpara et al., 2015). The genus *Photobacterium* (16%) is the second largest genus of the family, followed by *Aliivibrio* (3%).

**Figure 1:**
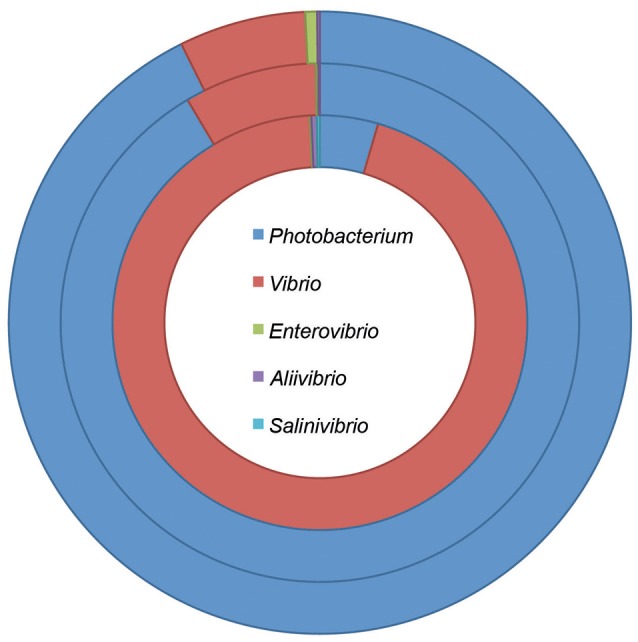
Taxonomic composition of the *Vibrionaceae* family at different pelagic depths. The data was retrieved from the Tara oceans project, and included stations where information was acquired for the three depths (Sunagawa et al., 2015). The rings represent the different depths: surface water layer—down to 5 m depth (outer ring); deep chlorophyll maximum layer—from 5 to 200 m depth (middle ring); and mesopelagic zone—from 250 to 1,000 m depth (inner ring).

Species belonging to the *Vibrionaceae* family are believed to be very similar (Vitulo et al., 2007). However, most studies have been based on *Vibrio* species, and only a few have included *Photobacterium* strains. Recent metagenomic data shows that although these two genera seem to have similar ecological strategies, *Photobacterium* spp. dominates in the lower pelagic depths (surface water layer and deep chlorophyll maximum layer) while *Vibrio* spp. is more prevalent in higher depths (mesopelagic zone) (Figure 1; Sunagawa et al., 2015). This suggests different ecological strategies and roles of the two genera.

*Photobacterium* includes species with different life-styles: free-living, symbiotic or pathogenic (Urbanczyk et al., 2011). The genus currently covers 28 species of which 26 have validly published names (www.bacterio.net) (“P. atrarenae” and “P. marinum” are not yet validly published names) (Gomez-Gil et al., 2011; Kim et al., 2011; Lucena et al., 2011; Urbanczyk et al., 2011; Srinivas et al., 2013; Figge et al., 2014; Liu et al., 2014; Lo et al., 2014; Moreira et al., 2014; Machado et al., 2015a). Of these 28, only 27 are available since no type strain was deposited for *P. aplysiae*, (Seo et al., 2005; Holmes and Farmer, 2008). Twenty-two of the 28 species have been described during the last 15 years, making it a relatively new genus.

The majority of *Photobacterium* strains have been isolated from marine environments, although isolation of a *P. halotolerans* from the rhizosphere of a terrestrial weed has recently been reported (Mathew et al., 2015a,b). All species of this genus were originally thought to be luminescent, but it has become clear that a large number of species are not luminescent (Lucena et al., 2011; Urbanczyk et al., 2011). This ecological important characteristic is part of the symbiotic life style of some *Photobacterium* species and is attributed to the expression of the *lux-rib* operon (Dunlap, 2009). This operon has been used taxonomically for the division of the genus into clades (Ast and Dunlap, 2004; Urbanczyk et al., 2011).

Recently, we and other scientists have sequenced the genomes of several strains of this genus, representing a total of 16 different species. Most of the strains have been isolated from the marine environment, although with different approaches and goals (Table S1). Some were isolated as symbionts of marine animals, others from spoiled fish, and others host-associated with oysters, crabs and fish (Ast et al., 2007; Gomez-Gil et al., 2011; Urbanczyk et al., 2011; Bjornsdottir-Butler et al., 2016). Some were isolated as free-living organisms from coastal or pelagic waters (Nogi et al., 1998), and *P. galatheae* S2753 was isolated due to its antagonism against pathogenic bacteria (Gram et al., 2010; Machado et al., 2015a).

The purpose of the present study was to use this recent genomic information to investigate the genomic diversity within the genus, and analyzed the possible genetic foundation of known physiological traits. These analyses allow a better understanding of the genus phylogeny by elucidating evolutionary relationships using genomic information; it reveals the genomic diversity existent within the genus and the role of foreign DNA in genomic diversity acquisition.

## Materials and methods

### General genome statistics

The shotgun whole genome sequences from 35 strains of the *Photobacterium* genus and the sequence of *Vibrio pacinii* DSM 19139 were used in this study (Table S1). Calculation of basic genome statistics such as size, GC content and amino acid usage was done using the CMG biotools (Vesth et al., 2013). These tools were also used for gene finding using prodigalrunner, generation of BLAST atlases comparing the protein-coding open reading frames and for the pan- and core-genome analyses of the studied strains.

### Phylogenetic analyses

The 16S rRNA gene sequences used are the publically available sequences originally published for each strain or obtained from the whole genome sequence when other sequence was not available. The gene sequences used for the MLSA (*ftsZ, gapA, gyrB, mreB, pyrH, recA, rpoA*) and the *fur* gene sequence, were obtained from the genomes using the CLC Main Workbench (CLC Aarhus, Denmark version 7) (Table S2). An annotation-based search was performed for the gene sequences of interest using the NCBI annotation. The genomes not annotated at NCBI were annotated using RAST (Aziz et al., 2008; Overbeek et al., 2014). The accession numbers for 16S rRNA gene sequences and the gene locus tags are provided in Table S2. The gene *topA* was not used in the MLSA, since no homologous sequence was found in the genome of *P. galatheae* S2753. Alignments and Maximum Likelihood Phylogeny trees were done using CLC Main Workbench. Maximum Likelihood Phylogeny trees were constructed using the Neighbor Joining method with the Jukes-Cantor nucleotide distance measure. The topology of the tree was tested with 1,000 bootstrap replications. MEGA 6 was used to finalize the tree design (Tamura et al., 2013).

The Genome-to-Genome Distance Calculator by DSMZ was used to determine *in silico* DNA-DNA re-association percent values (Auch et al., 2010), and the Average Nucleotide Identity (ANI) calculator to estimate the ANI values (Goris et al., 2007). The nucleotide identity percentages for the *fur* gene and the MLSA were calculated using the pair-wise comparison tool of CLC Main Workbench.

### *lux-rib* operon

The *lux* genes were identified by homology search to previously described genes (Ast and Dunlap, 2004; Urbanczyk et al., 2008, 2012) using protein sequence BLAST tools of CLC Main Workbench with identity/query coverage cutoff of 50/50. Identified *lux* operons were then blasted against whole genome sequences of the studied *Photobacterium* strains using MultiGeneBLAST (Medema et al., 2013).

### Identification of prophages, genomic islands and secondary metabolite clusters

Prophages, genomic islands and secondary metabolite clusters were identified using the online tools PHAST, IslandViewer 3 and antiSMASH 3.0, respectively (Zhou et al., 2011; Dhillon et al., 2015; Weber et al., 2015). The whole genome sequences were submitted to the different tools, and the identified clusters compared using MultiGeneBLAST (Medema et al., 2013).

### CRISPR-*cas*

Genome sequences were analyzed in CRISPRfinder (Grissa et al., 2007) and homology searches for CRISPR associated genes were performed using CLC Main Workbench. Using the CLC Main Workbench BLAST tool, direct repeats and protospacers were compared among each other and to previously identified prophages. Confirmation of *cas* genes was performed by BLAST comparison of the identified open-reading frames to the NCBI nucleotide database.

### Plasmid comparison and virulence genes

We used previously described *Photobacterium* plasmids to search the genomes for contigs belonging to plasmids using CLC Main Workbench. Plasmids used in the search included: pPHDD1 (FN597600.2), pAQU1 (AB571865.1), pP99-018 (AB277723.1), pP91278 (AB277724.1), pPHDP60 (KC344732.1), pPHDP10 (DQ069059.1), pPHDP70 (KP100338.1), pP9014 (AB453229.1), pPH1 (AY789019.1), pPBPR1 (CR377818.1), and the unnamed plasmid from *P. gaetbulicola* Gung47 (KC687076.1).

### Identification of virulence, histamine production, and transposase genes

The genes *hlyA* and *dly* are key virulence genes of *P. damselae* (Rivas et al., 2011, 2013; Le Roux et al., 2015). Virulence related genes were identified using the BLAST tools of CLC Main Workbench with protein identity/query coverage cutoff of 50/50. Also the genetic basis for histamine production was evaluated, by searching for genes related to histamine production, previously identified in *Photobacterium* strains (Bjornsdottir-Butler et al., 2016). An annotation based identification of transposase genes was performed in CLC Main Workbench.

### Singleton analysis

The number of singletons per genome was calculated using the EDGAR platform for comparative genomics (Blom et al., 2009). A private project has been created by uploading the whole genome sequences of the studied *Photobacterium* strains and the analysis performed using default settings for singleton identification.

## Results and discussion

### Phylogeny

The 16S rRNA gene phylogeny is widely used in the classification of *Photobacterium* species and it is sometimes the only phylogenetic discrimination provided when new species are described (Park et al., 2006; Rivas et al., 2006; Gomez-Gil et al., 2011; Liu et al., 2014). However, this gene has low discriminatory power in resolving species relatedness; for instance, *P. angustum* strains appear scattered throughout the phylogenetic tree, and *P. damselae* subsp. *piscicida* DI21 and “P. marinum” AK15 cluster closely to *V. pacinii* DSM18139 (Figure S1).

**Figure 2:**
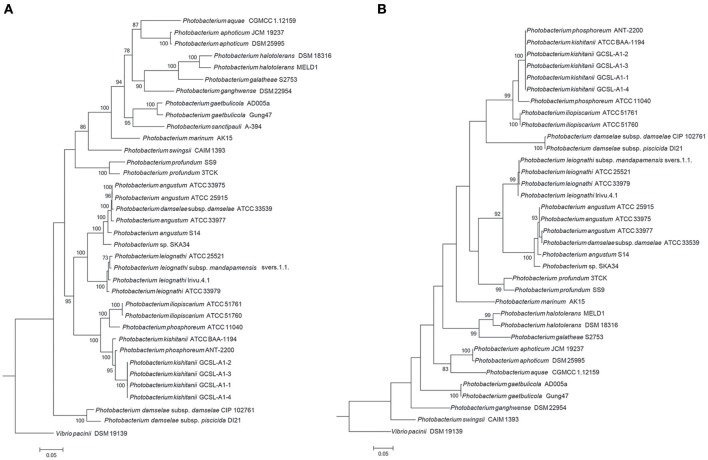
Phylogenetic trees. The trees were constructed using seven house-keeping genes (*ftsZ, gapA, gyrB, mreB, pyrH, recA, rpoA*) **(A)** and the *fur* gene **(B)**. The topology of the tree was tested with 1,000 bootstrap replications, and *Vibrio pacinii* DSM 19139 used to root the tree.

The limitations of the use of the 16S rRNA gene as a phylogenetic marker in *Vibrionaceae* have been previously reported (Sawabe et al., 2007; Machado and Gram, 2015), and the use of Multi-Locus Sequence Analysis (MLSA) has been proposed when evaluating *Vibrionaceae* phylogenetic relationships (Thompson F. L. et al., 2005; Sawabe et al., 2007, 2013; Pascual et al., 2010; Gabriel et al., 2014). Here, we used several clustering methods to investigate the phylogenetic relationships within the *Photobacterium* genus. Different approaches (MLSA, *fur*, amino-acid usage, average nucleotide identity [ANI]) led to distinct phylogenetic results; nevertheless the core phylogenetic groups identified were the same (Figure 2, Figures S2, S3).

In the MLSA, sequences of seven genes (*ftsZ, gapA, gyrB, mreB, pyrH, recA, rpoA*) allowed a phylogenetic evaluation using a concatenation length of 7,230 bp. Here, strains belonging to the same species cluster tightly. The same was observed for the recently described identification marker, the ferric up-take regulator (*fur*) gene (Machado and Gram, 2015). The *fur* gene has been described as a powerful identification marker in the *Vibrionaceae* family, however the phylogenetic relationships observed were different from the ones obtained using MLSA and whole genome sequence methods (amino-acid usage and ANI) (Figure 2, Figures S2, S3).

The phylogenetic analysis showed a misidentification of two isolates, *P. phosphoreum* ANT-2200 and *P. damselae* subsp. *damselae* ATCC 33539. Based on our results, *P. phosphoreum* ANT-2200 should be classified as *P. kishitanii*, as has previously been suggested (Ast and Dunlap, 2004; Urbanczyk et al., 2011; Bjornsdottir-Butler et al., 2016), and was here confirmed by the phylogenetic assessment and the genomic analyses. The genome identified as belonging to *P. damselae* subsp. *damselae* ATCC 33539 was similar to the ones of *P. angustum*, but the 16S rRNA gene phylogeny using the original sequence placed this strain elsewhere. In fact, partial (FJ971859) and complete (NR_040831) 16S rRNA sequences available for strain ATCC 33539 presented 52 and 68% of query coverage, respectively, with 94 and 96% of identity to the whole genome sequence identified as belonging to *P. damselae* subsp. *damselae* ATCC33539. Since no other physiological discrepancies have been previously reported, we believe that the case of *P. damselae* subsp. *damselae* ATCC 33539 might be a wrong whole genome sequence submission to NCBI or sequencing of a wrong strain.

This study did not clarify the phylogenetic association of *P. damselae* as MLSA, *fur*, ANI, amino-acid usage analyses placed it in different branches, confirming its unstable association (Lucena et al., 2011). In contrast, although the species “P. marinum” has not been recognized, we here show that the type-strain AK15 seems to be indeed the representative of a new species (Srinivas et al., 2013).

In order to provide a quantitative evaluation of the different phylogenetic approaches, *in silico* DNA-DNA hybridization, *fur* and MLSA percentage of identity values were calculated and correlated to the ANI. Comparing all the tested phylogenetic methods, resulted in correlation coefficients between 0.84 and 0.95 (Figure S4). Interestingly, the *fur* gene of approximately 450 bp correlated better with the ANI than the MLSA, which used approximately 6,800 bp more in the analysis. This strengthens the previous suggestion of the use of the *fur* gene in the classification of *Vibrionaceae* family strains (Machado and Gram, 2015). We therefore suggest the use of *fur* analysis in the future classification of new *Photobacterium* isolates, although MLSA should still be used to evaluate evolutionary relationships.

The division of the *Photobacterium* genus into two or three clades has been previously suggested (Lucena et al., 2011; Urbanczyk et al., 2011). The most recent review on this genus proposed two clades, based both on the molecular phylogenetic evaluation, but also on the creation of a luminous/symbiotic and a non-luminous/non-symbiotic clusters (Urbanczyk et al., 2011). Our results support the phylogenetic relationship previously observed between the species of each cluster, but the identification of genes responsible for luminescence suggest that *P. angustum* and *P. damselae* are not luminescent species as reported before (Urbanczyk et al., 2011; Figure S5). Some strains of *P. damselae* have acquired *lux* genes through horizontal gene transfer (HGT) (Urbanczyk et al., 2008), which might explain the absence of these genes in the studied strains. On the other hand, luminescence of *P. angustum* strains varies (Urbanczyk et al., 2011), but we could not identify *lux* genes in any of the six *P. angustum* strains. Furthermore, *P. ganghwense* has been defined as bioluminescent (Park et al., 2006); however, it has not been described how this feature was evaluated in the original study, nor could we identify *lux* genes in the genome of the type-strain. Therefore, the generic division of luminous vs. non-luminous clades should be avoided.

### Genomic diversity

#### General genomic features

**Figure 3:**
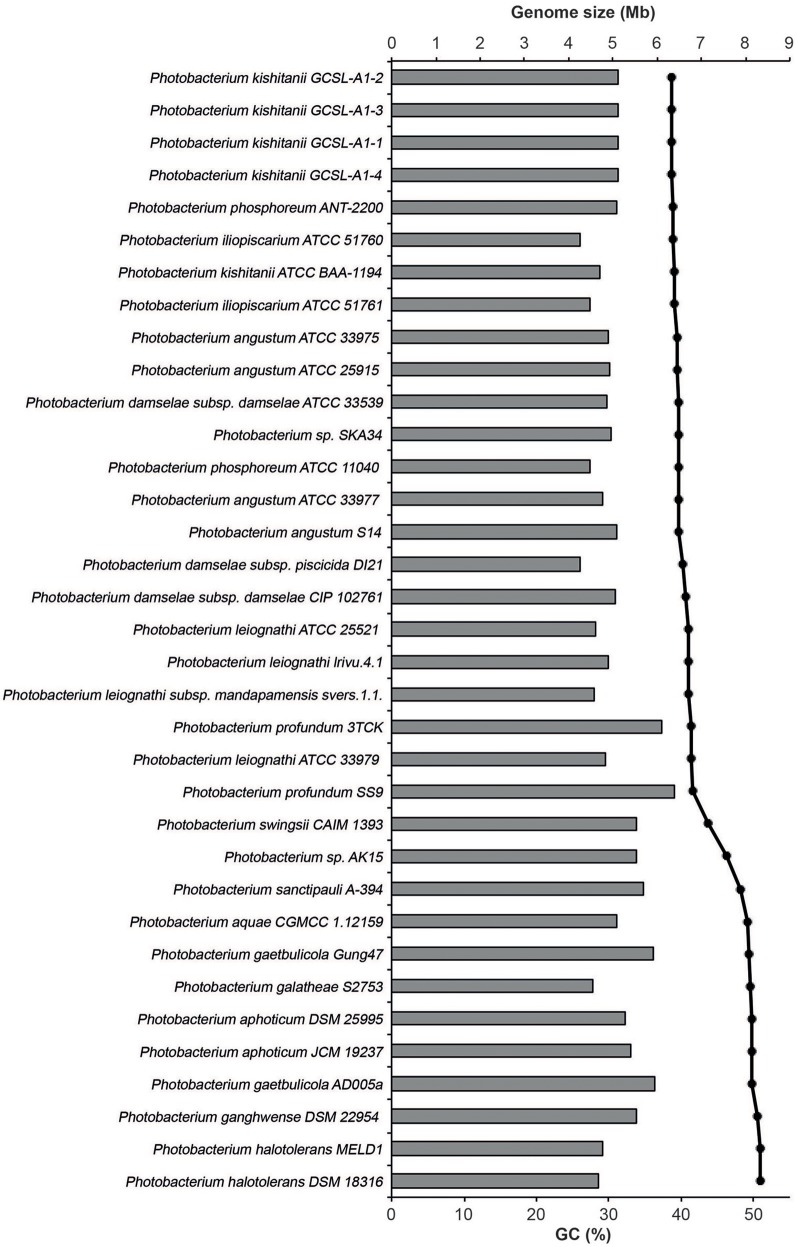
Genome size and GC content of the studied genomes. The size is represented in mega-bases (Mb) by the horizontal bars and the percentage of GC by the black dots.

The *Photobacterium* genomes ranged in size from 4.2 to 6.4 Mb (Figure 3). The genome size range was in accordance with the ones reported for other genera from the *Vibrionaceae* family (Thompson et al., 2009). The largest genomes found belonged to *P. profundum*, which is an extremely versatile species. *P. profundum* SS9 is able to grow at cold temperatures and in pressurized environments (Eloe et al., 2008; Lauro et al., 2008, 2014). It seems therefore logical that such environmental versatility comes associated with a larger genome size (Konstantinidis and Tiedje, 2004).

The GC content of the genomes varied between 38.7 and 50.9%, and the strains clustered in two groups based on GC%: one of approximately 40% and another of approximately 50%. The only exceptions were *P. swingsii* CAIM1393 and *Photobacterium* sp. AK15, which had a GC content of 43.4 and 46.2%, respectively. The GC content has been linked to a division of strains that also is reflected in their environmental conditions as well as to amino acid usage (Lightfield et al., 2011). Independently of the method used, the species with higher GC content clustered together, suggesting an evolutionary association of GC content and phylogenetic proximity within the genus (Figure 2).

Several theories for genome evolution in bacteria explaining smaller cell size and genomes, the different GC content, the genomic reductions and expansions have been presented and discussed (Moran et al., 2007; Hunt et al., 2008a; Newton et al., 2010; Morris et al., 2012; Fernández-Gómez et al., 2013; Giovannoni et al., 2014; Luo and Moran, 2015). It has, for example, been shown that genome size and GC content are related with the ecological strategies of the different marine bacteria, with free-living bacteria having lower GC content and smaller genomes, as compared to patch-associated bacteria (Luo and Moran, 2015). Also, it is expected that symbionts, parasites and commensals would experience genome reduction due to specialization (Morris et al., 2012; Giovannoni et al., 2014). Here, we observe that the smallest genomes and lower GC content are indeed found in the known symbiotic organisms (*P. iliopiscarium, P. damselae, P. phosphoreum*), but also in *P. galatheae* and *P. halotolerans*. This could be explained by genetic drift and streamlining, respectively (Giovannoni et al., 2014; Luo and Moran, 2015).

The only two closed genomes (*P. profundum* SS9 and *P. gaetbulicola* Gung47) have a larger and a smaller chromosome of approximately 4 and 2 Mb, plus megaplasmids of 80 and 35 Kb, respectively. The presence of 2 chromosomes is a trend of the *Vibrionaceae* family and it is assumed that the draft genomes used also include two chromosomes and large plasmids, but fully closed genomes would be required to assert this. The number of genes per genome was estimated using prodigalrunner and ranged from 4,041 to 7,027.

**Figure 4:**
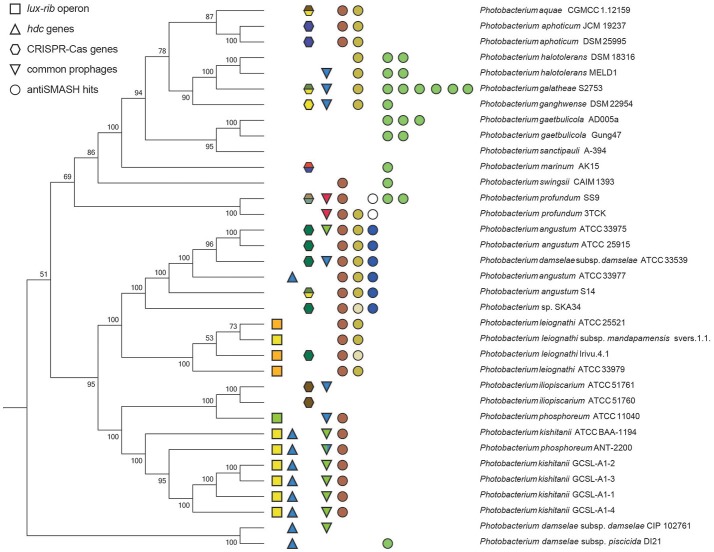
Summary figure of some of the shared genomic traits between the *Photobacterium* strains. The phylogenetic tree is based on the MLSA phylogenetic distances. Squares represent the *lux-rib* operon, color-coded for the different gene cluster architectures. Triangles indicate the presence of genes coding for histamine producing proteins. Hexagons indicate the presence of CRISPR-Cas loci, color-coded for the different gene cluster architectures. Inverted triangles indicate the common prophages, each different also color-coded. Circles represent antiSMASH hits: brown for aryl-polyene biosynthetic gene cluster; yellow for ectoine biosynthetic gene cluster; blue for terpene biosynthetic gene cluster; white for polyunsaturated fatty acid biosynthesis gene cluster; and green for NRPS biosynthetic gene clusters.

To visualize the protein-coding gene content conservation in the *Photobacterium* genus, we constructed a BLAST atlas using *P. profundum* SS9 and *P. gaetbulicola* Gung47 as reference genomes (Figure S6). As previously described for other members of the *Vibrionaceae* family (Thompson et al., 2009), the *Photobacterium* large chromosome seems to be more conserved between species than the smaller chromosome or plasmids (Figure S6). The smaller chromosomes are highly variable and harbor the main genomic differences between strains of the same species. These secondary chromosomes and plasmids therefore seem to be the source of the genetic plasticity, mirrored in the different phenotypes observed within members of the *Photobacterium* genus and *Vibrionaceae* family in general (Vesth et al., 2010; Lukjancenko and Ussery, 2014). A well-studied example is *V. splendidus*, where the genomes of strains from the same species vary and result in distinct phenotypic capabilities (Thompson J. R. et al., 2005; Hunt et al., 2008a). This diversity is also evident for the *Photobacterium* species in the several genetic features analyzed, such as virulence, bioluminescence, histamine production, secondary metabolism and CRISPR-Cas operons. All these genomic features presented random distributions rarely correlated with phylogenetic relatedness (Figure 4), raising the issue of species-phenotype association within the *Photobacterium* genus. Nevertheless, genomic features such as genome size and GC content seem to be associated with different lifestyles adopted by *Photobacterium* species.

Bioluminescence for example was initially thought to be a widespread feature of the genus (Urbanczyk et al., 2011). We identified the genetic basis for bioluminescence only in three species *P. kishitanii, P. phosphoreum*, and *P. leiognathi* (Figure S5), isolated from different marine animals (Table S1). This also compared with the phylogenetic analysis (Figure 2A), with the exception of *P. iliopiscarium*, that may have lost this trait due to niche adaptation (Figure 4). Analysis of the *lux-rib* operon further showed that strains ATCC 25521, ATCC 33979 and *Irivu*.4.1 are most likely *P. leiognathi* subsp. *leiognathi*, according to their *lux-rib* gene organization (Figure S5) (Ast and Dunlap, 2004). Similarly, histamine production seems to be specific for *P. kishitanii* and *P. damselae*, however genes responsible for this feature could be identified also in one *P. angustum* strain (ATCC 33977) (Figure S7). Scombrotoxin fish poisoning (high levels of histamine) is the most frequent cause of fish poisoning incidents within the United States (Pennotti et al., 2013). Recently, high-histamine producing *Photobacterium* strains have been isolated from freshly caught fish (Bjornsdottir-Butler et al., 2016), raising awareness for the need to revise food safety rules regarding sea food. There are also several reports describing *P. phosphoreum* as histamine producing species (Kanki et al., 2004, 2007), but we did not identify the genes responsible for histamine production in *P. phosphoreum* ATCC 11040, although it may be present in other *P. phosphoreum* strains.

#### Pan- and core-genome

**Figure 5:**
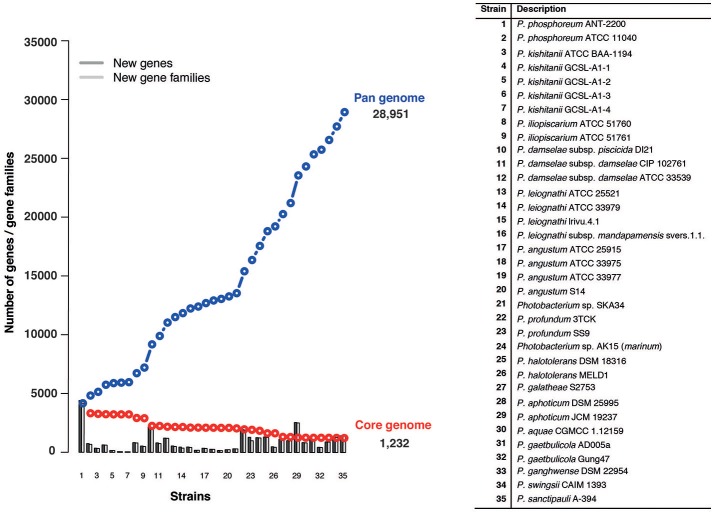
Pan- and core-genome plot of the 35 *Photobacterium* strains. The pan- and core-genome calculation was performed using protein sequence BLAST with identity/query coverage cutoff of 50/50. Proteins matching were considered the same gene family. Proteins present in all the tested genomes were considered part of the core-genome.

The pan-genome refers to the total number of genes in all the 35 strains, while the core genome represents the number of orthologous genes shared between them. Using 35 genomes we identified a pan-genome of 28,951 genes and a core-genome of 1,232 genes (Figure 5). Taking into consideration the average gene number of 4,750 for the *Photobacterium* strains, 1,232 genes, represents approximately 25% of the total genome, meaning that approximately 1/4 of the genome is conserved in all the strains. The number of core-genome genes is in agreement with what has been found in other marine *Gammaproteobacteria* (Qin et al., 2013), although this is two times higher than what has been found for the *Vibrio* genus, where the core-genome comprised approximately 500 genes (Thompson et al., 2009). This difference can be explained by the high number of *Vibrio* species (more than 120), which reflects the genomic and ecological diversity of the genus.

The pan-genome for the *Photobacterium* genus alone is greater than what has been reported in a study of the *Vibrionaceae* family, where 43 genomes of a total of 13 species from three different genera were used (Thompson et al., 2009). This suggests *Photobacterium* harbors high genomic diversity, reflected in their ability to colonize different environmental niches (Konstantinidis and Tiedje, 2004; Konstantinidis et al., 2009) and supports the theory that high gene content variation exists in environmental marine strains (Tettelin et al., 2008; Konstantinidis et al., 2009). Using a power-law regression, it is possible to evaluate the openness of a pan-genome (Tettelin et al., 2008). The *Photobacterium* pan-genome is open, with a γ parameter of 0.62 in a power-law regression fitting relatively well the data analyzed (*R*^2^ = 0.89) (Figure S8). This genetic variation and uniqueness of each strain is also evident in the number of singletons per strain, which for some strains represent almost 20% of the genes identified in the genome (Table S3).

#### Plasmids and virulence genes

Most *Photobacterium* species have been described as symbiotic or associated with other marine organisms (Ast et al., 2007; Dunlap, 2009; Gomez-Gil et al., 2011; Urbanczyk et al., 2011, 2013). Furthermore, some strains of *P. damselae* are pathogens of marine organisms, especially fish (Hundenborn et al., 2013; Andreoni and Magnani, 2014). The key virulence genes of *P. damselae* are a phospholipase-D damselysin gene (*dly*) and a pore-forming toxin gene (*hlyA*), which act in a synergistic manner (Rivas et al., 2011, 2013; Le Roux et al., 2015). We conducted a homology search to evaluate the possible virulence of other *Photobacterium* species. The genes could only be identified in *P. damselae* subsp. *damselae* CIP 102761. In this strain, two copies of the *hlyA* gene were identified, one in contig_1, close to an IS4 transposase and a phage integrase and the other in contig_4, next to the *dly* gene.

Often draft-whole genome sequences contain plasmid sequences. Plasmids are important mediators of several physiological traits of *Photobacterium*, such as virulence, drug resistance and biosynthetic capabilities (Kim et al., 2008; Rivas et al., 2011; Nonaka et al., 2012; Osorio et al., 2015). We compared known *Photobacterium* plasmids to the here studied genomes. Plasmid pPHDD1 showed high similarity to contig_4 of *P*. *damselae* subsp. *damselae* CIP 102761 genome. Some of the contigs of *P. damselae* subsp. *piscicida* DI21 also had high similarity to plasmids pPHDP60, pPHDP10 and pPHDP70, which have been previously isolated from this strain (Osorio et al., 2008, 2015).

### Evidence of horizontal gene transfer (HGT)

The genetic exchange of foreign DNA by means of transposable elements, phage infection or conjugative plasmids has been suggested as a driving force in the evolution of members of *Vibrionaceae* (Reen et al., 2006; Vitulo et al., 2007; Gu et al., 2009; Lilburn et al., 2010; Urbanczyk et al., 2011). HGT can occur by uptake of environmental DNA, conjugative plasmids and bacteriophage infection. In the *Photobacterium* genus, studies on HGT are limited to the *lux-rib* operon, and a *chitinase A* and deoxyribodipyrimidine photolyase genes (Urbanczyk et al., 2008, 2012; Hunt et al., 2008b; Lauro et al., 2014) Here, we searched for evidence of genomic exchange by identifying prophages, transposases, CRISPR-Cas systems, genomic islands and secondary metabolite biosynthetic gene clusters.

#### Genomic islands and transposases

**Figure 6:**
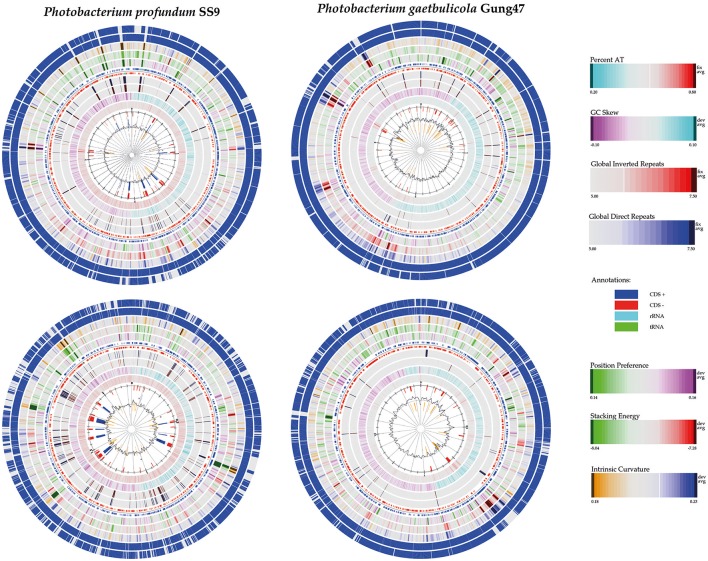
Identification of genomic islands in the fully sequenced genomes of *P. profundum* SS9 and of *P. gaetbulicola* Gung47. The genomes of the mentioned strains are compared to the genome of another strain of the same species, *P. profundum* 3CTK and *P. gaetbulicola* AD005, respectively. The circle inside the BLAST atlas shows the Island Viewer results (integrated results in red, SIGI-HMM in orange and IslandPath-DIMOB in blue).

Using Island Viewer (Dhillon et al., 2015), we searched for genomic islands in the fully sequenced genomes of *P. profundum* SS9 and *P. gaetbulicola* Gung47, and compared these to genomes of other strains from the same species (Figure 6). Some of the major genetic differences between the strains of the same species seem indeed to be related to the presence or absence of specific genomic islands. Genomic regions only present in the reference strain are placed close to identified genomic islands.

Additionally, strains varied in their number of transposase genes (Table S3). While *P. profundum* SS9 had 219 transposase genes, *P. leioghnathi* subsp. *mandapamensis svers*.1.1. had none. No correlation between number of prophages and number of transposase genes was observed. A high number of transposase genes could indicate transposon-mediated exchange of genetic material from any source (both plasmid-borne and random environmental DNA). *P. profundum* SS9 and *Photobacterium* sp. SKA34 are cases where transposase genes represent almost 4% of the total genes in the genome (Table S3). This is in accordance with metagenomics data that showed that transposase coding genes are the most abundant genes in nature, most likely accelerating biological diversification and evolution (Aziz et al., 2010) and that a higher number of transposase genes is present in higher oceanic depths (Konstantinidis et al., 2009).

#### Prophages and CRISPR-Cas systems

Using the PHAge Search Tool (PHAST) (Zhou et al., 2011) we identified 33 intact prophage sequences and 72 incomplete ones (Table S3). From the 33 intact ones, 17 were unique, while the other 16 were re-occurrences of three prophages. One was present only in *P. profundum* strains; another in all *P. kishitanii* strains, plus one *P. damselae* and one *P. angustum*; and the third was randomly distributed (Figure 4).

**Figure 7:**
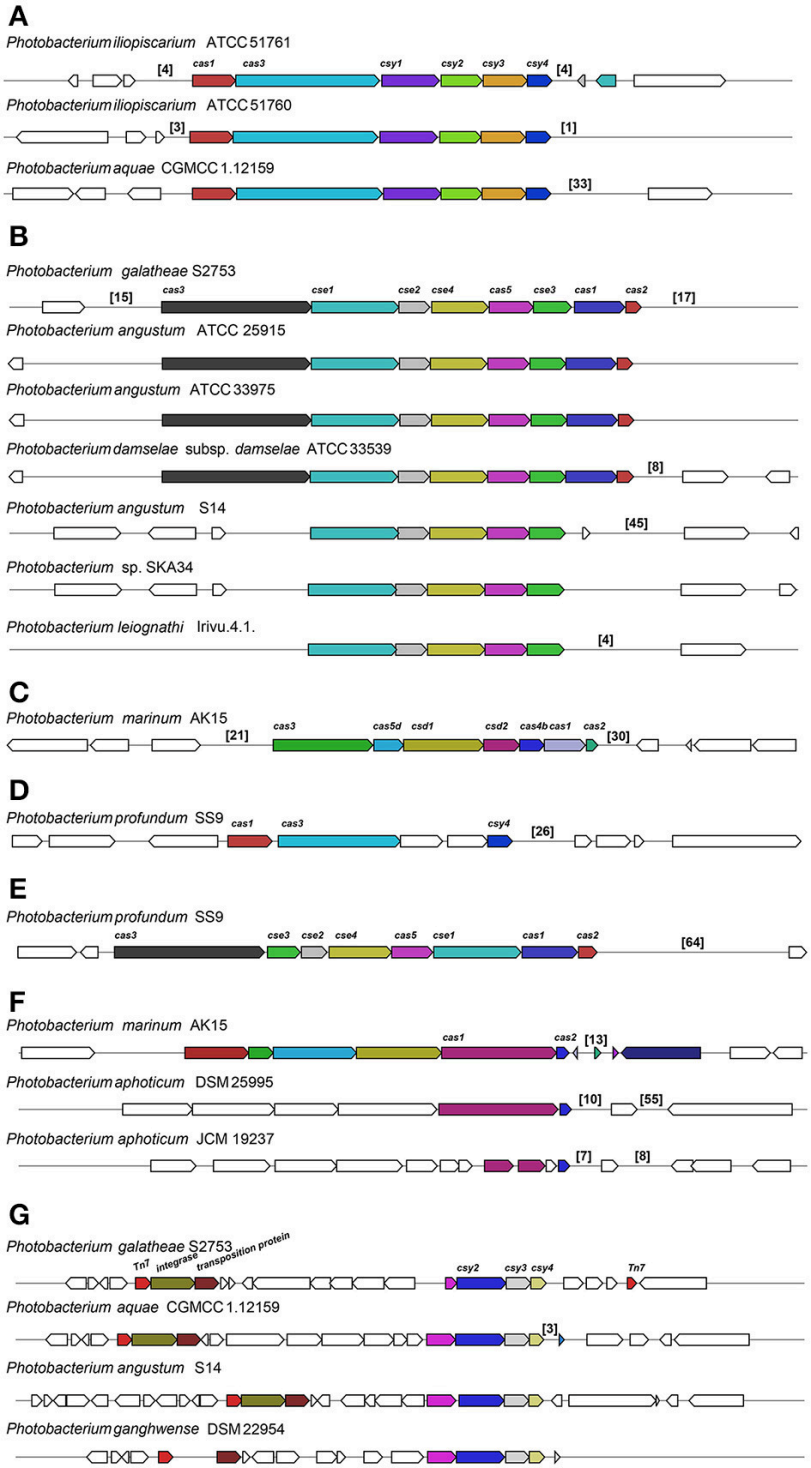
Different CRISPR/Cas subtypes identified across the *Photobacterium* genomes. CRIPR/Cas similar to *Yersinia pestis* **(A)**, *Escherichia coli* **(B)** and *Desulfovibrio vulgaris* **(C)**, and other gene organizations **(D–G)**. The numbers in brackets correspond to the number of protospacers identified in that region.

The bacterial and archaeal adaptive immune systems entail CRISPR-Cas modules (Makarova et al., 2015) and we queried the genomes for the architecture of the CRISPR-Cas systems including the *cas* gene organization, the direct repeats (DR) and the protospacers in the CRISPR locus. We divided the architecture of the CRISPR-Cas systems into seven clusters (Figure 7). Most of the architectures identified were similar to the ones described in *Yersinia pestis, Escherichia coli* and *Desulfovibrio vulgaris* (Figures 7A–C; Haft et al., 2005). Also, we identified two clusters in *P. profundum* SS9 similar to the ones of *Y. pestis* and *E. coli*, encoded in the chromosome and plasmid, respectively (Figures 7D,E). These clusters had different gene arrangement and/or included genes coding for unknown proteins in the operon. Two other clusters containing CRISPR-associated genes were identified (Figures 7F,G).

The estimation of DRs and protospacers in the CRISPR arrays is very difficult in draft genomes due to the short sequencing reads and the repetitive nature of the sequences, which make them difficult to assemble correctly. In most of the cases, both direct repeats and protospacers could be identified both upstream and downstream of the *cas*-operon. These were also identified elsewhere in the genome or in distinct contigs, usually consisting of one of the ends of the contig. For example, further upstream from the Tn7 in the *P. galatheae* S2753 (Figure 7G), an array of 16 protospacers could be identified beside a gene coding for a DNA nicking enzyme.

The DRs that flanked the protospacers were extracted and compared. The similarity of the DRs correlated with the type of clusters the strain had. DRs from clusters (a) and (d) were similar, as were the ones within (b) and (f). The protospacers are short sequences that are derived from bacteriophages or other foreign DNA, such as conjugative plasmids (Attar, 2015; Makarova et al., 2015). Therefore, these sequences may provide a history of encounters of a specific bacterium with phages and/or plasmids. The number of spacers varied considerable between different strains, from 1 to 64 protospacers in the same CRISPR array. Arrays with extensive number of protospacers in some strains indicate numerous bacteriophage infections, which is the case of e.g., *P. profundum* SS9, *P. angustum* S14 and *P. aquae* CGMCC 1.12159 with 64, 45, and 33 protospacers, respectively (Figure 7). The 4 spacers in *P. leiognathi* lrivu.4.1 were 100% identical to the first 4 spacers in *P. angustum* S14, which had an array of 45 spacers. Also high similarity (>93% Identity) could be identified in the first spacers of *P. angustum* ATCC 25915, ATCC 33975 and *P. damselae* subsp. *damselae* ATCC 33539 (which should be classified as *P. angustum*), although for the *P. angustum* strains the protospacers have not just been identified downstream of the *cas* operon, but elsewhere.

We have also compared the protospacers to the prophage sequences identified in these genomes. The bacterial immune system (CRISPR-Cas) allows the protection against re-infection by the same bacteriophage (Attar, 2015), nevertheless, for *P. angustum* ATCC 33975 an array of 9 sequential spacers were 100% identical to an intact prophage sequence identified within the same genome, suggesting multiple re-infection events.

Phage infection seems to be a frequent event in some strains, supported by the extensive number of protospacers in the CRISPR arrays and the number of prophages within the genomes (Figure 7). Remarkably, the *cas* operons previously associated with *E. coli, D. vulgaris* and *Y. pestis* were identified in *Photobacterium* strains (Haft et al., 2005). Yet, presence/absence and type of cluster seems randomly spread across the different species (Figure 4).

#### Secondary metabolism

Another indication of high genomic exchange is the number and distribution of secondary metabolism biosynthetic clusters, many of which are believed to be acquired by HGT (Khaldi et al., 2008). Secondary metabolites such as non-ribosomal peptides and polyketides are known to have antagonistic properties, which may constitute an advantage in several ecological niches. Although *Photobacterium* strains have mostly been studied due to their association with marine animals, their potential in drug discovery and other applications has recently been reported (Wietz et al., 2010b; Machado et al., 2014, 2015a,b; Nielsen et al., 2014; Mathew et al., 2015a,b).

Using antiSMASH 3.0 (Weber et al., 2015), different biosynthetic gene clusters were identified (Table S4) with some clusters being present across the genus and others being species specific (Figure 4). The same terpene biosynthetic cluster could be identified in all the *P. angustum* strains (including the misidentified *P. damselae* subsp. *damselae* ATCC 33539). The function of this terpene cluster is not known, but it may be related to the planktonic lifestyle of this species, since all the *P. angustum* strains were isolated from seawater. Another species-specific cluster was the polyunsaturated fatty-acid (PUFA) cluster present in *P. profundum* strains. This cluster could be involved in the high pressure and cold temperature adaptation of this species, since these PUFAs are known to modify membrane fluidity in response to hydrostatic pressure and temperature (Campanaro et al., 2005).

Other biosynthetic clusters such as siderophore, aryl-polyene and ectoine were widely distributed across the genus (Figure 4). A siderophore cluster was present in 11 strains, although two distinct siderophore biosynthetic clusters were identified. The ectoine cluster was present in 18 strains (two had only two out of three genes needed for its biosynthesis). The most widely distributed cluster was the aryl-polyene biosynthetic cluster, identified in 23 out of the 35 strains. Interestingly strains associated with marine animals did not have a siderophore cluster nor were they prolific in other secondary metabolite clusters (Figure 4, Figure S4). Strains isolated from seawater or sediments seem to be enriched in secondary metabolite clusters, when compared to marine animal associated species. These strains have therefore the genetic capability of possibly antagonizing microbial competitors in their environment.

The distribution of secondary metabolite clusters suggests events of gain and loss of these clusters throughout the evolution of *Photobacterium* species. The random presence of specific traits, i.e., not phylogenetically related, has been reported for other genus. For example, in *Pseudovibrio* the random distribution of type IV secretion systems was attributed to the frequency with which those genes are horizontally acquired (Cascales and Christie, 2003; Romano et al., 2016). In fact, several of the interesting phenotypic traits identified in *Photobacterium* strains, such as bioluminescence, virulence, histamine production and piezophilia seem to be acquired by HGT (Ast and Dunlap, 2004; Campanaro et al., 2005; Dunlap, 2009; Urbanczyk et al., 2011; Hundenborn et al., 2013; Andreoni and Magnani, 2014; Osorio et al., 2015; Bjornsdottir-Butler et al., 2016).

## Conclusion

Here, we focused on the genomic diversity within the marine genus *Photobacterium*. We found a high genomic diversity within this genus, and some genomic traits appear to be related to lifestyle, such as GC content, genome size, bioluminescence, secondary metabolism and virulence. We evaluated genomic traits related to genomic exchange such as prophage infection, presence of genomic islands and genes coding for transposases and show that these are abundant in the *Photobacterium* genus, indicating numerous genomic changes throughout evolution of the genus. Genomic exchange might therefore be the strongest driver in the genomic evolution of this genus, reflected in the different lifestyles of the species.

## Author contributions

HM designed the study, performed the analysis and interpreted the results. HM and LG wrote the manuscript. Both authors read and approved the final manuscript.

### Conflict of interest statement

The authors declare that the research was conducted in the absence of any commercial or financial relationships that could be construed as a potential conflict of interest.
