# Supplementary material for: Comparative Genomics Reveals High Genomic Diversity in the Genus Photobacterium
Source: Front Microbiol. 2017 Jun 29;8:1204. doi: 10.3389/fmicb.2017.01204 (PMC5489566; doi:10.3389/fmicb.2017.01204)
Supplement: Supplementary file 1 [file Table1.PDF]

Table S1 – Strains used in this study, accession numbers of its genomes and isolation source. ND – not determined.

Strain	WGS/ChrI	ChrII	Plasmid	Isolation Source	References
Photobacterium angustum ATCC 25915	JZSO01			seawater	(Bjornsdottir-Butler *et al.*, 2016)
Photobacterium angustum ATCC 33975	JZSM01			seawater	(Bjornsdottir-Butler *et al.*, 2016)
Photobacterium angustum ATCC 33977	JZSN01			seawater	(Bjornsdottir-Butler *et al.*, 2016)
Photobacterium angustum S14	AAOJ01			surface waters	(Lauro *et al.*, 2009)
Photobacterium aphoticum DSM 25995	LDOV01			water	(Machado *et al.*, 2015)
Photobacterium aphoticum JCM 19237	BBMN01			water samples (coral reef vicinity)	(Al-saari *et al.*, 2014)
Photobacterium aquae CGMCC 1.12159	LDOT01			mariculture system	(Machado *et al.*, 2015)
Photobacterium damselae subsp. *damselae* ATCC 33539	JZSI01			damselfish skin ulcers	(Bjornsdottir-Butler *et al.*, 2016)
Photobacterium damselae subsp. *damselae* CIP 102761	ADBS01			damselfish skin ulcers	
Photobacterium damselae subsp. *piscicida* DI21	AKYG01		KC344732	liver of fish with pasteurellosis	
Photobacterium gaetbulicola AD005a	JWLZ01			coastal marine water	
Photobacterium gaetbulicola Gung47	CP005973	CP005974	KC687076	tidal flat	
Photobacterium galatheae S2753	JMIB01			surface of a mussel	(Machado *et al.*, 2014)
Photobacterium ganghwense DSM 22954	LDOU01			water	(Machado *et al.*, 2015)
Photobacterium halotolerans DSM 18316	AULG01			water samples	
Photobacterium halotolerans MELD1	JWYV01			roots of *Phragmites australis*	(Mathew *et al.*, 2015)
Photobacterium iliopiscarium ATCC 51760	JZSQ01			herring pyloric ceca	(Bjornsdottir-Butler *et al.*, 2016)
Photobacterium iliopiscarium ATCC 51761	JZSR01			salmon pyloric ceca	(Bjornsdottir-Butler *et al.*, 2016)
Photobacterium kishitanii ATCC BAA-1194	JZSP01			marine fish	(Bjornsdottir-Butler *et al.*, 2016)
Photobacterium kishitanii GCSL-A1-1	JZTE01			albacore tuna-anal vent	(Bjornsdottir-Butler *et al.*, 2016)

Photobacterium kishitanii GCSL-A1-2	JZTD01			albacore tuna-anal vent	(Bjornsdottir-Butler *et al.*, 2016)
Photobacterium kishitanii GCSL-A1-3	JZTC01			albacore tuna-anal vent	(Bjornsdottir-Butler *et al.*, 2016)
Photobacterium kishitanii GCSL-A1-4	JZTB01			albacore tuna-anal vent	(Bjornsdottir-Butler *et al.*, 2016)
Photobacterium leiognathi ATCC 25521	JZSK01			fish in the family leiognathidae	(Bjornsdottir-Butler *et al.*, 2016)
Photobacterium leiognathi ATCC 33979	JZSL01			ND	(Bjornsdottir-Butler *et al.*, 2016)
Photobacterium leiognathi lrivu.4.1	BANQ01			light organ of *Equulites rivulatus*	(Urbanczyk *et al.*, 2013)
Photobacterium leiognathi subsp. *mandapamensis* svers.1.1	BACE01			light organ of *Siphamia versicolor*	(Urbanczyk *et al.*, 2011)
Photobacterium phosphoreum ANT-2200	CCAR01			water column (2,200 m depth)	
Photobacterium phosphoreum ATCC 11040	JZSJ01			ND	(Bjornsdottir-Butler *et al.*, 2016)
Photobacterium profundum 3TCK	AAPH01			shallow water	(Urbanczyk *et al.*, 2013)
Photobacterium profundum SS9	CR354531	CR354532	CR377818	deep-sea sediment	(Vezzi, 2005)
Photobacterium sanctipauli A-394	JGVO01			bleached *Madracis decactis* (*Scleractinia*)	(Moreira *et al.*, 2014)
Photobacterium sp. AK15 (*marinum*)	AMZO01			sediment	
Photobacterium sp. SKA34	AAOU01			surface waters	(Persson *et al.*, 2009)
Photobacterium swingsii CAIM 1393	LELC01			hemolymph of *Maja brachydactyla*	(Gomez-Gil *et al.*, 2011)

Al-saari, N., Meirelles, P. M., Mino, S., Suda, W., Oshima, K., Hattori, M., *et al.* (2014). Draft Genome Sequences of Two *Vibrionaceae* Species, *Vibrio ponticus* C121 and *Photobacterium aphoticum* C119, Isolated as Coral Reef Microbiota. *Genome Announc.* 2, e01095-14. doi:10.1128/genomeA.01095-14.

Bjornsdottir-Butler, K., McCarthy, S., Dunlap, P. V., and Benner, R. a. (2016). *Photobacterium angustum* and *Photobacterium kishitanii* : Psychrotrophic High Histamine-Producing Bacteria Indigenous to Tuna. *Appl. Environ. Microbiol.*, AEM.02833-15. doi:10.1128/AEM.02833-15.

Gomez-Gil, B., Roque, A., Rotllant, G., Peinado, L., Romalde, J. L., Doce, A., *et al.* (2011). *Photobacterium swingsii* sp. nov., isolated from marine organisms. *Int J Syst Evol Microbiol* 61, 315–319. doi:10.1099/ijs.0.019687-0.

Lauro, F. M., McDougald, D., Thomas, T., Williams, T. J., Egan, S., Rice, S., *et al.* (2009). The genomic basis of trophic strategy in marine bacteria. *Proc. Natl. Acad. Sci. U. S. A.* 106, 15527–15533. doi:10.1073/pnas.0903507106.

Machado, H., Giubergia, S., Mateiu, R. V., and Gram, L. (2015). *Photobacterium galatheae* sp. nov., a bioactive bacterium isolated from a mussel in the Solomon Sea. *Int. J. Syst. Evol. Microbiol.* 65, 4503–4507. doi:10.1099/ijsem.0.000603.

Machado, H., Månsson, M., and Gram, L. (2014). Draft genome sequence of *Photobacterium halotolerans* S2753, producer of bioactive secondary metabolites. *Genome Announc.* 2, 9–10. doi:10.1128/genomeA.00535-14.Copyright.

Mathew, D. C., Mathew, M., Gicana, G., and Huang, C. (2015). Genome Sequence of *Photobacterium halotolerans* MELD1 , with Mercury Reductase (*merA*), Isolated from *Phragmites australis*. *Genome Announc.* 3, 12204. doi:10.1128/genomeA.00530-15.Copyright.

Moreira, A. P. B., Duytschaever, G., Chimetto Tonon, L. a, Fróes, A. M., de Oliveira, L. S., Amado Filho, G., *et al.* (2014). *Photobacterium sanctipauli* sp. nov. isolated from bleached *Madracis decactis* (Scleractinia) in the St Peter & St Paul Archipelago, Mid-Atlantic Ridge, Brazil. *PeerJ* 2, e427. doi:10.7717/peerj.427.

Persson, O. P., Pinhassi, J., Riemann, L., Marklund, B. I., Rhen, M., Normark, S., *et al.* (2009). High abundance of virulence gene homologues in marine bacteria. *Environ. Microbiol.* 11, 1348–1357. doi:10.1111/j.1462-2920.2008.01861.x.

Urbanczyk, H., Ogura, Y., Hendry, T. A., Gould, A. L., Kiwaki, N., Atkinson, J. T., *et al.* (2011). Genome sequence of *Photobacterium mandapamensis* strain svers.1.1, the bioluminescent symbiont of the cardinal fish *Siphamia versicolor*. *J. Bacteriol.* 193, 3144–3145. doi:10.1128/JB.00370-11.

Urbanczyk, H., Urbanczyk, Y., Hayashi, T., and Ogura, Y. (2013). Diversification of two lineages of symbiotic *Photobacterium*. *PLoS One* 8, 1–9. doi:10.1371/journal.pone.0082917.

Vezzi, A. (2005). Life at Depth: *Photobacterium profundum* genome sequence and expression analysis. *Science (80-.).* 307, 1459–1461. doi:10.1126/science.1103341.
