# Supplementary material for: Comparative Genomics Reveals High Genomic Diversity in the Genus Photobacterium
Source: Front Microbiol. 2017 Jun 29;8:1204. doi: 10.3389/fmicb.2017.01204 (PMC5489566; doi:10.3389/fmicb.2017.01204)
Supplement: Supplementary file 2 [file Table2.PDF]

Table S2 – Genes used in the MLSA analyses. RAST indicates that genes were annotated and identified using RAST annotations otherwise the locus_tag is indicated. When multiple locus_tag are indicated, the one used is in bold.

**Strain**	***16S rRNA***	***ftsZ***	***gapA***	***gyrB***	***mreB***	***pyrH***	***recA***	***rpoA***	***topA***	***fur***
P. damselae **subsp.** ***damselae*** **CIP 102761**	AB032015	VDA_001180	VDA_001240	VDA_003250	VDA_003504	VDA_001360	VDA_001282	VDA_003464	VDA_002384	VDA_002511
P. damselae **subsp.** ***piscicida*** **DI21**	RAST	RAST	RAST	RAST	RAST	RAST	RAST	RAST	RAST	RAST
P. damselae **subsp.** ***damselae*** **ATCC 33539**	**NR_040831**; NR_119052.1; FJ971859	UB36_13705	UB36_17335	UB36_18700	UB36_13420	UB36_20750	UB36_17065	UB36_20875	UB36_04120	UB36_04870
Photobacterium **sp. SKA34**	AF255623	SKA34_22402	SKA34_22052	SKA34_17333	SKA34_22729	SKA34_21349	SKA34_21755	SKA34_22977	SKA34_03419	SKA34_02659
Photobacterium **sp. AK15 (marinum)**	NR_133050.1	C942_04822	C942_01118	C942_02909	C942_04764	C942_03492	C942_01172	C942_02953	C942_01398	C942_01251
P. profundum **3TCK**	DQ027054.1	P3TCK_24370	P3TCK_24736	P3TCK_22929	P3TCK_24070	P3TCK_25440	P3TCK_25016	P3TCK_23870	P3TCK_27904	P3TCK_16014
P. profundum **SS9**	**AB003191**; PSU91586	PBPRA3211	PBPRA3132	PBPRA0011	PBPRA3270	PBPRA2966	PBPRA3068	PBPRA0345	PBPRA2477	PBPRA1035
P. leiognathi **subsp.** ***mandapamensis*** **svers.1.1.**	KC456610.1	RAST	RAST	RAST	RAST	RAST	RAST	RAST	RAST	RAST
P. leiognathi **lrivu.4.1**	**NR_115541.1**; AY204498.1	RAST	RAST	RAST	RAST	RAST	RAST	RAST	RAST	RAST
P. leiognathi **ATCC 25521**	D25309.1	UB42_05010	UB42_10705	UB42_10125	UB42_05295	UB42_15885	UB42_10975	UB42_19320	UB42_03440	UB42_15530
P. leiognathi **ATCC 33979**	-	UB34_03160	UB34_06890	UB34_12385	UB34_02875	UB34_16825	UB34_07160	UB34_19695	UB34_11980	UB34_13820
P. angustum **S14**	AJ630163.2	VAS14_19911	VAS14_20231	VAS14_22357	VAS14_19626	VAS14_20966	VAS14_20496	VAS14_19336	VAS14_18414	VAS14_17676
P. angustum **ATCC 25915**	NR_119046.1	UB33_14340	UB33_16680	UB33_17120	UB33_14050	UB33_19385	UB33_16410	UB33_20160	UB33_12670	UB33_04410
P. angustum **ATCC 33977**	-	UB35_14360	UB35_16015	UB35_16905	UB35_14070	UB35_19055	UB35_15750	UB35_20175	UB35_01635	UB35_02395
P. angustum **ATCC 33975**	AY900628.1	UB39_10920	UB39_15480	UB39_18015	UB39_10630	UB39_17475	UB39_15210	UB39_20870	UB39_10245	UB39_00400
P. halotolerans **DSM 18316**	NR_042975.1	RAST	RAST	RAST	RAST	RAST	RAST	RAST	RAST	RAST
P. galatheae **S2753**	**KR704916**; FJ457476	EA58_05035	EA58_04580	EA58_08470	EA58_05325	EA58_18525	EA58_04325	EA58_08130	-	EA58_03265
P. halotolerans **MELD1**	KC903134.1	KY46_10315	KY46_09915	KY46_18430	KY46_10600	KY46_18145	KY46_19655	KY46_21110	KY46_05665	KY46_04475
P. phosphoreum **ANT-2200**	EU881910.1	PPBDW_I20087	PPBDW_I20168	PPBDW_I10065	PPBDW_I20029	PPBDW_I20304	PPBDW_I20220	PPBDW_I10338	PPBDW_I21735	PPBDW_50029
P. phosphoreum **ATCC 11040**	**NR_119047.1**; NR_115205.1; AY341437.1	UB41_05570	UB41_09335	UB41_11210	UB41_12100	UB41_17225	UB41_09585	UB41_19395	UB41_00885	UB41_01500
P. gaetbulicola **Gung47**	**NR_117271.1;** GQ260188.1	H744_2c0707	H744_2c0784	H744_2c0322	H744_2c0648	H744_2c0925	H744_2c0841	H744_2c0600	H744_2c2703	H744_2c1241

***P. gaetbulicola* AD005a**	RJ45_01325	RJ45_05475	RJ45_25915	RJ45_14550	RJ45_16640	RJ45_03805	RJ45_17415	RJ45_17760	RJ45_22920	RJ45_13565
***P. sanctipauli* A-394**	**NR_126301.1**; KF748537.1; NR_126275.1; KC751088.1	RAST	RAST	RAST	RAST	RAST	RAST	RAST	RAST	RAST
***P. swingsii* CAIM 1393**	**NR_117351.1;** GQ386822.1	AB733_1352 5	AB733_21045	AB733_18230	AB733_13235	AB733_17490	AB733_21315	AB733_22840	AB733_00535	AB733_10315
***P. ganghwense* DSM 22954**	**NR_043295.1**; AY960847.2	ABT57_1962 5	ABT57_18865	ABT57_22530	ABT57_19910	ABT57_00220	ABT57_19135	ABT57_10545	ABT57_01030	ABT57_2390 0
***P. aquae* CGMCC 1.12159**	**NR_133815.1**; JQ948040.1	ABT56_0925 5	ABT56_07995	ABT56_20835	ABT56_08970	ABT56_03810	ABT56_07730	ABT56_21155	ABT56_02290	ABT56_0305 5
***P. kishitanii* GCSL-A1-3**	UA42_22830	UA42_10690	UA42_16515	UA42_17650	UA42_10405	UA42_17110	UA42_16770	UA42_22285	UA42_03665	UA42_04315
***P. kishitanii* GCSL-A1-2**	UA40_22770	UA40_10935	UA40_16310	UA40_17100	UA40_11220	UA40_20535	UA40_16565	UA40_22275	UA40_05810	UA40_06525
***P. kishitanii* ATCC BAA-1194**	**NR_042852.1**; AY341439.1	UB40_07970	UB40_14090	UB40_16315	UB40_08255	UB40_19380	UB40_14340	UB40_20590	UB40_01490	UB40_00845
***P. kishitanii* GCSL-A1-1**	UA41_22745	UA41_06365	UA41_15730	UA41_16960	UA41_06650	UA41_20485	UA41_15985	UA41_22160	UA41_03300	UA41_03950
***P. kishitanii* GCSL-A1-4**	UA38_22550	UA38_09245	UA38_14910	UA38_15685	UA38_08960	UA38_19805	UA38_15165	UA38_21845	UA38_12435	UA38_05395
***P. iliopiscarium* ATCC 51761**	AY849432.1	UB37_10495	UB37_13050	UB37_09940	UB37_11510	UB37_11190	UB37_17965	UB37_18570	UB37_01590	UB37_13790
***P. iliopiscarium* ATCC 51760**	**NR_111990.1**; AY643710.1; NR_043067.1	UB38_13970	UB38_10860	UB38_13175	UB38_12625	UB38_12205	UB38_10615	UB38_18020	UB38_01660	UB38_01080
***P. aphoticum* JCM 19237 (C119)**	JCM19237_74 8	JCM19237_2 573	JCM19237_4 425	JCM19237_6 778, JCM19237_6 779	JCM19237_2 485, JCM19237_2 486	JCM19237_8 80	JCM19237_4 500	JCM19237_7 18	JCM19237_4 151	JCM19237_5 54
***P. aphoticum* DSM 25995**	FN796493.1	ABT58_0204 0	ABT58_19985	ABT58_01355	ABT58_02325	ABT58_18090	ABT58_19735	ABT58_21575	ABT58_14605	ABT58_1373 0
***Vibrio pacinii* DSM 19139**	AJ316194	DQ907366.1	DQ907300.1	AB298264.1	KP635265.1	AJ842486.1	AJ580850.1	AJ842674.1	DQ907508.1	BS19_RS178 60
