# Supplementary material for: Comparative Genomics Reveals High Genomic Diversity in the Genus Photobacterium
Source: Front Microbiol. 2017 Jun 29;8:1204. doi: 10.3389/fmicb.2017.01204 (PMC5489566; doi:10.3389/fmicb.2017.01204)
Supplement: Supplementary file 3 [file Table3.PDF]

Table S3 - Number of prophages, transposases and singletons identified in each of the genomes.

	Strain	Prophages		Prodigal Gene count	Number of transposase	Transposase genes (%)	Number of singletons	Singletons (%)
		Intact	Incomplete					
**1**	Photobacterium damselae subsp. damselae CIP 102761	3	2	4443	41	0.92	259	5.83
**2**	Photobacterium damselae subsp. piscicida DI21	1	1	4830	5	0.10	250	5.18
**3**	Photobacterium damselae subsp. damselae ATCC 33539	1	0	4605	4	0.09	62	1.35
**4**	Photobacterium sp. SKA34	0	6	4505	160	3.55	155	3.44
**5**	Photobacterium sp. AK15	1	0	4865	2	0.04	826	16.98
**6**	Photobacterium profundum 3TCK	1	0	5455	9	0.16	495	9.07
**7**	Photobacterium profundum SS9	2	3	5828	219	3.76	546	9.37
**8**	Photobacterium leiognathi subsp. mandapamensis svers.1.1.	1	2	4041	0	0.00	101	2.50
**9**	Photobacterium leiognathi lrivu.4.1	0	2	4342	13	0.30	222	5.11
**10**	Photobacterium leiognathi ATCC 25521	0	1	4251	33	0.78	93	2.19
**11**	Photobacterium leiognathi ATCC 33979	0	2	4360	20	0.46	155	3.56
**12**	Photobacterium angustum S14	0	3	4507	23	0.51	180	3.99
**13**	Photobacterium angustum ATCC 25915	0	3	4562	3	0.07	73	1.60
**14**	Photobacterium angustum ATCC 33977	0	1	4389	5	0.11	74	1.69
**15**	Photobacterium angustum ATCC 33975	1	0	4698	3	0.06	67	1.43
**16**	Photobacterium halotolerans DSM 18316	0	1	4257	3	0.07	339	7.96
**17**	Photobacterium galatheae S2753	3	0	4110	14	0.34	816	19.85
**18**	Photobacterium halotolerans MELD1	1	0	4322	54	1.25	252	5.83
**19**	Photobacterium phosphoreum ANT-2200	2	1	4401	3	0.07	428	9.73
**20**	Photobacterium phosphoreum ATCC 11040	1	2	4206	36	0.86	110	2.62
**21**	Photobacterium gaetbulicola Gung47	1	10	5226	43	0.82	300	5.74
**22**	Photobacterium gaetbulicola AD005a	0	8	5335	16	0.30	312	5.85
**23**	Photobacterium sanctipauli A-394	1	2	5996	1	0.02	662	11.04
**24**	Photobacterium swingsii CAIM 1393	0	2	5487	9	0.16	468	8.53
**25**	Photobacterium ganghwense DSM 22954	3	1	5024	13	0.26	485	9.65
**26**	Photobacterium aquae CGMCC 1.12159	0	2	4687	18	0.38	457	9.75
**27**	Photobacterium kishitanii GCSL-A1-3	2	0	4742	12	0.25	2	0.04
**28**	Photobacterium kishitanii GCSL-A1-2	2	0	4801	12	0.25	2	0.04
**29**	Photobacterium kishitanii ATCC BAA-1194	1	0	4283	2	0.05	66	1.54
**30**	Photobacterium kishitanii GCSL-A1-1	2	0	4811	13	0.27	5	0.10
**31**	Photobacterium kishitanii GCSL-A1-4	2	0	4658	11	0.24	4	0.09
**32**	Photobacterium iliopiscarium ATCC 51761	0	0	4230	42	0.99	260	6.15
**33**	Photobacterium iliopiscarium ATCC 51760	0	7	4254	29	0.68	102	2.40
**34**	Photobacterium aphoticum JCM 19237	1	2	7027	1	0.01	573	8.15
**35**	Photobacterium aphoticum DSM 25995	0	8	4673	18	0.39	207	4.43
	**Total**	**33**	**72**		**890**		**9408**	
