# Supplementary material for: Comparative Genomics Reveals High Genomic Diversity in the Genus Photobacterium
Source: Front Microbiol. 2017 Jun 29;8:1204. doi: 10.3389/fmicb.2017.01204 (PMC5489566; doi:10.3389/fmicb.2017.01204)
Supplement: Supplementary file 4 [file Table4.PDF]

Table S4 – AntiSMASH results for each of the strains.

Strain	1	2	3	4	5	6	7	8	9	10	11	12	13
**P. angustum ATCC 25915**	Arylpolyene-Ectoine	Terpene	Bacteriocin	Siderophore									
**P. angustum ATCC 33975**	Siderophore	Bacteriocin	Terpene	Ectoine-Arylpolyene									
**P. angustum ATCC 33977**	Bacteriocin	Arylpolyene-Ectoine	Terpene	Siderophore									
**P. angustum S14**	Terpene	Arylpolyene-Ectoine	Siderophore	Bacteriocin									
**P. aphoticum DSM 25995**	Arylpolyene	Ectoine	Bacteriocin	Other	Siderophore								
**P. aphoticum JCM 19237**	Bacteriocin	Arylpolyene	Ectoine										
**P. aquae CGMCC 1.12159**	Bacteriocin	Other	Ectoine	Arylpolyene									
**P. damselae subsp. damselae ATCC 33539**	Bacteriocin	Siderophore	Terpene	Arylpolyene-Ectoine									
**P. damselae subsp. damselae CIP 102761**													
**P. damselae subsp. piscicida DI21**	Nrps-T1pks												
**P. gaetbulicola AD005a**	Nrps-T1pks	Nrps-T1pks	Bacteriocin	Bacteriocin	Nrps-Transatpks	Other							
**P. gaetbulicola Gung47**	Bacteriocin	Bacteriocin	Transatpks-Nrps	Other	Nrps-T1pks								
**P. galatheae S2753**	Other	Nrps	Other	Bacteriocin	Nrps-T1pks	Nrps	Siderophore	T1pks	Other	Ectoine	Nrps	Nrps	Nrps
**P. ganghwense DSM 22954**	Ectoine	Arylpolyene-Ectoine	T1pks	Nrps	Bacteriocin	Other							
**P. halotolerans DSM 18316**	Nrps	Siderophore	Ectoine	Nrps									
**P. halotolerans MELD1**	Bacteriocin	Nrps	Ectoine	Siderophore	Nrps	Otherks-Butyrolactone							
**P. iliopiscarium ATCC 51760**	Bacteriocin												
**P. iliopiscarium ATCC 51761**	Bacteriocin												
**P. kishitanii ATCC BAA-1194**	Bacteriocin	Arylpolyene											
**P. kishitanii GCSL-A1-1**	Bacteriocin	Arylpolyene											
**P. kishitanii GCSL-A1-2**	Bacteriocin	Arylpolyene											
**P. kishitanii GCSL-A1-3**	Bacteriocin	Arylpolyene											

**P. kishitanii GCSL-A1-4**	Bacteriocin	Arylpolyene				
**P. leiognathi ATCC 25521**	Bacteriocin	Ectoine-Arylpolyene				
**P. leiognathi ATCC 33979**	Bacteriocin	Ectoine-Arylpolyene				
**P. leiognathi lrivu.4.1**	Bacteriocin	Arylpolyene				
**P. leiognathi subsp. mandapamensis svers.1.1.**	Bacteriocin	Ectoine-Arylpolyene				
**P. phosphoreum ANT-2200**	Bacteriocin	Arylpolyene				
**P. phosphoreum ATCC 11040**	Bacteriocin	Arylpolyene				
**P. profundum 3TCK**	Arylpolyene	Bacteriocin	Ectoine	Pufa-Otherks	Bacteriocin	
**P. profundum SS9**	Pufa-Otherks	Nrps-T1pks	Bacteriocin	Other	Arylpolyene	Nrps
**P. sanctipauli A-394**	Bacteriocin					
**Photobacterium sp. AK15 (marinum)**	Other	Nrps-T1pks	Bacteriocin			
**Photobacterium sp. SKA34**	Terpene	Arylpolyene	Siderophore	Siderophore		
**P. swingsii CAIM 1393**	Nrps	Arylpolyene	Siderophore	Bacteriocin		
