# Supplementary figures and images for: Comparative Genomics Reveals High Genomic Diversity in the Genus Photobacterium
Source: Front Microbiol. 2017 Jun 29;8:1204. doi: 10.3389/fmicb.2017.01204 (PMC5489566; doi:10.3389/fmicb.2017.01204)

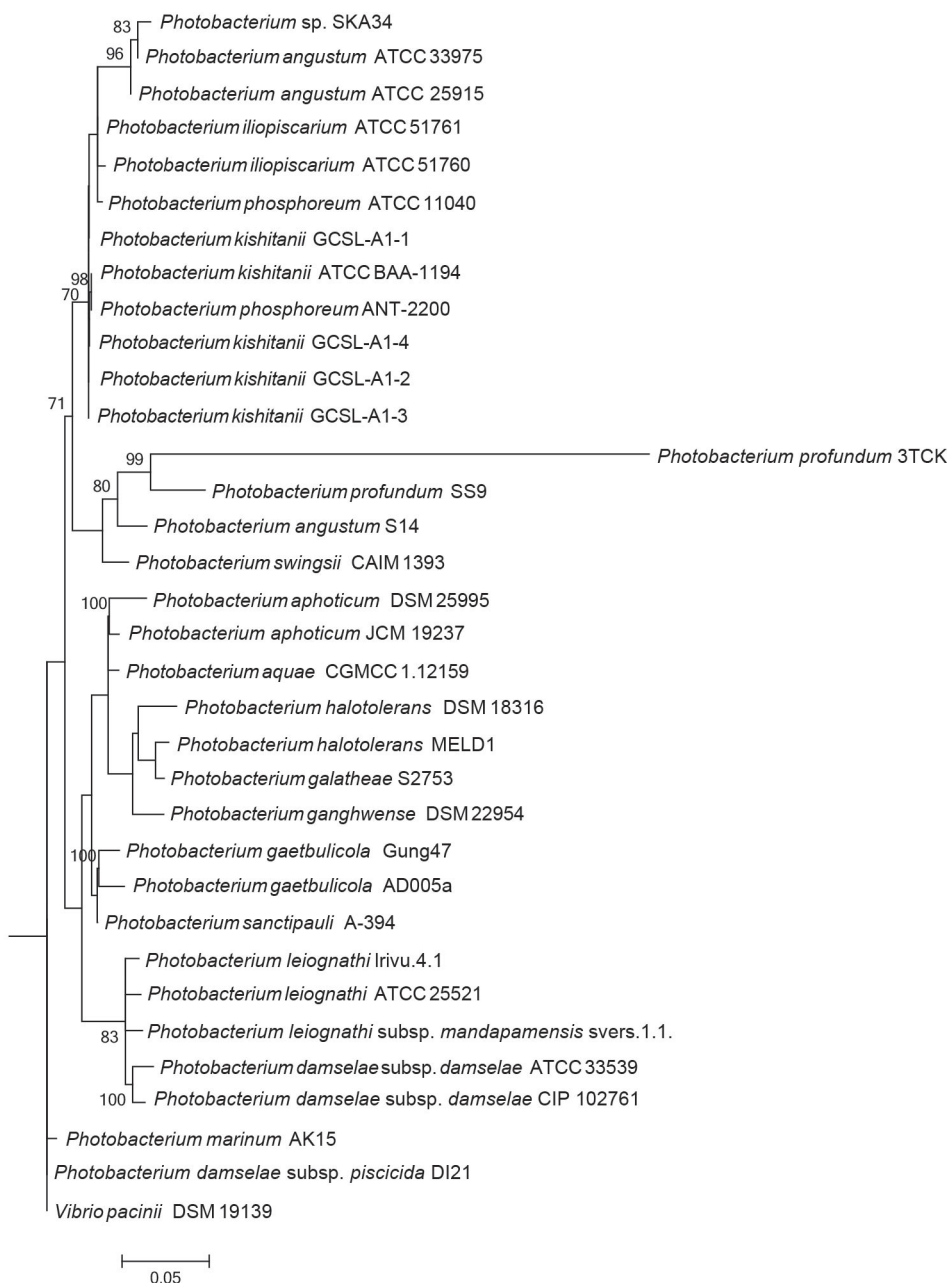

**Figure S1.** Phylogenetic tree using the 16S rRNA gene sequences.

Supplement: Supplementary file 5 [file Image1.PDF]

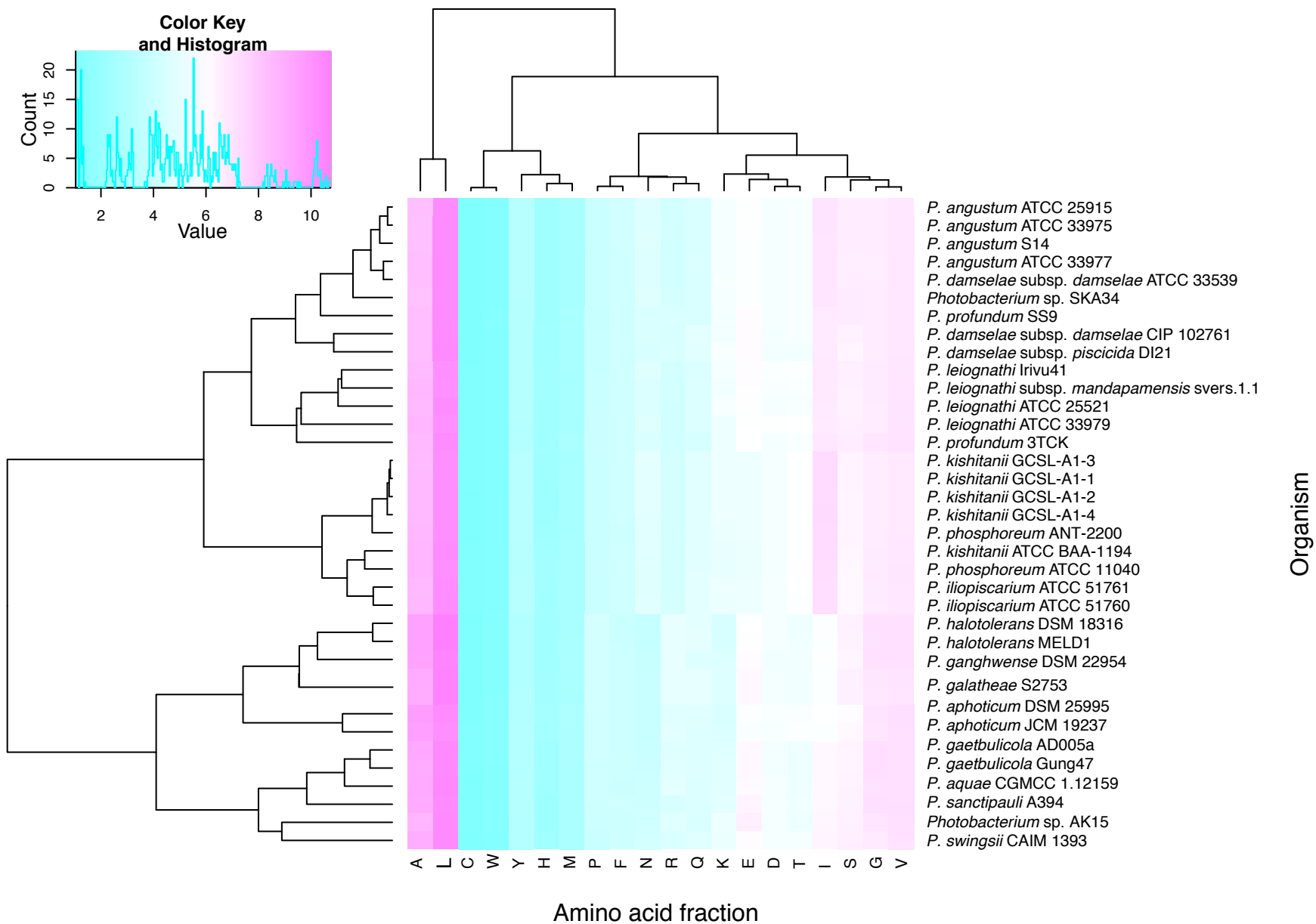

**Figure S2.** Amino-acid usage.

Supplement: Supplementary file 6 [file Image2.PDF]

**Figure S3. Average Nucleotide Identity heatmap.**

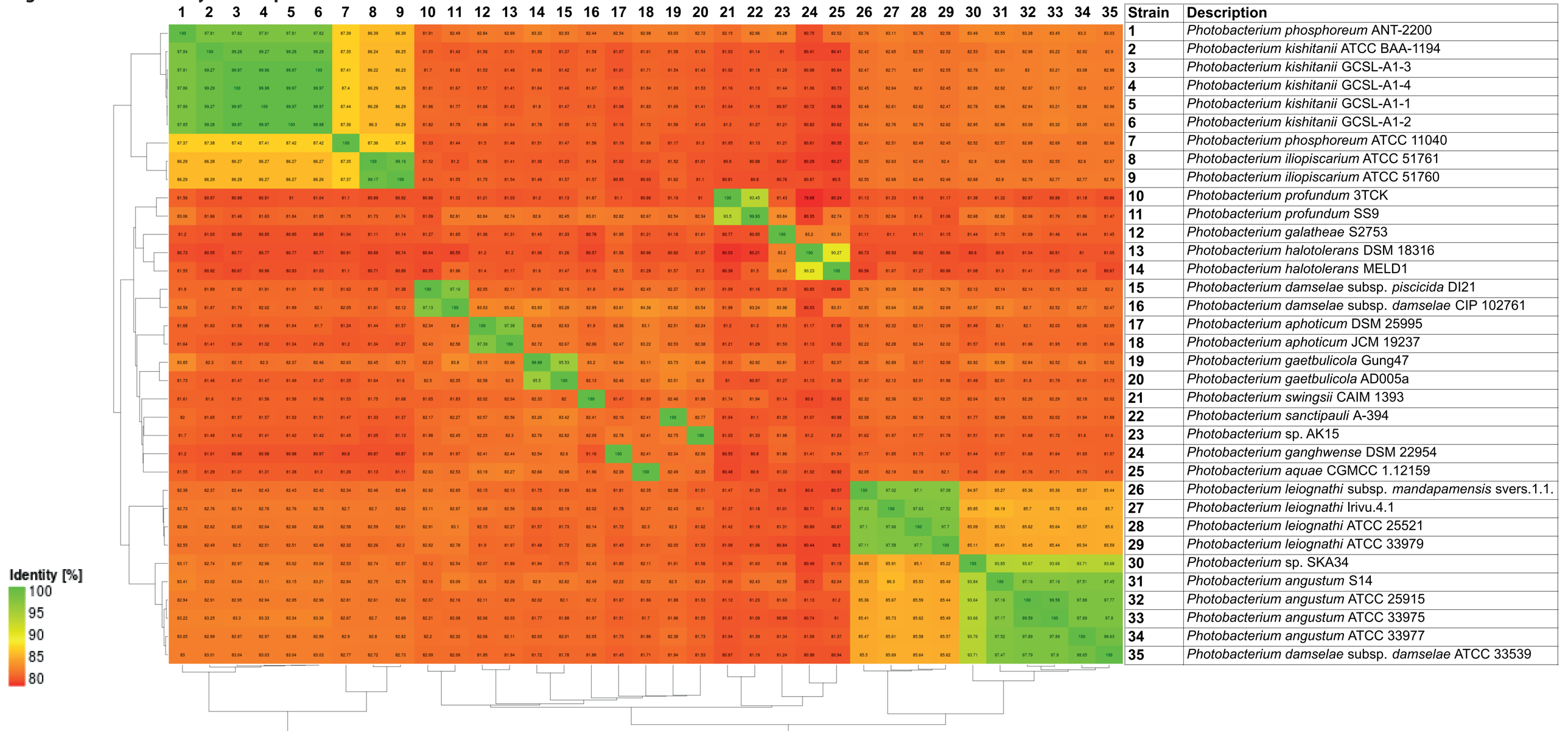

Supplement: Supplementary file 7 [file Image3.PDF]
