# Supplementary material for: Comparative Genomics Reveals High Genomic Diversity in the Genus Photobacterium
Source: Front Microbiol. 2017 Jun 29;8:1204. doi: 10.3389/fmicb.2017.01204 (PMC5489566; doi:10.3389/fmicb.2017.01204)

Figure S4. Evaluation of the discriminatory power of the different methods.
Assessment was made using the genomic information of *P. angustum* ATCC 33975 in comparison with the genomic information from all the other *Photobacterium* strains used in the study.
The values were plotted and the equation of correlation is presented.
The R^2 represents the correlation coefficient.

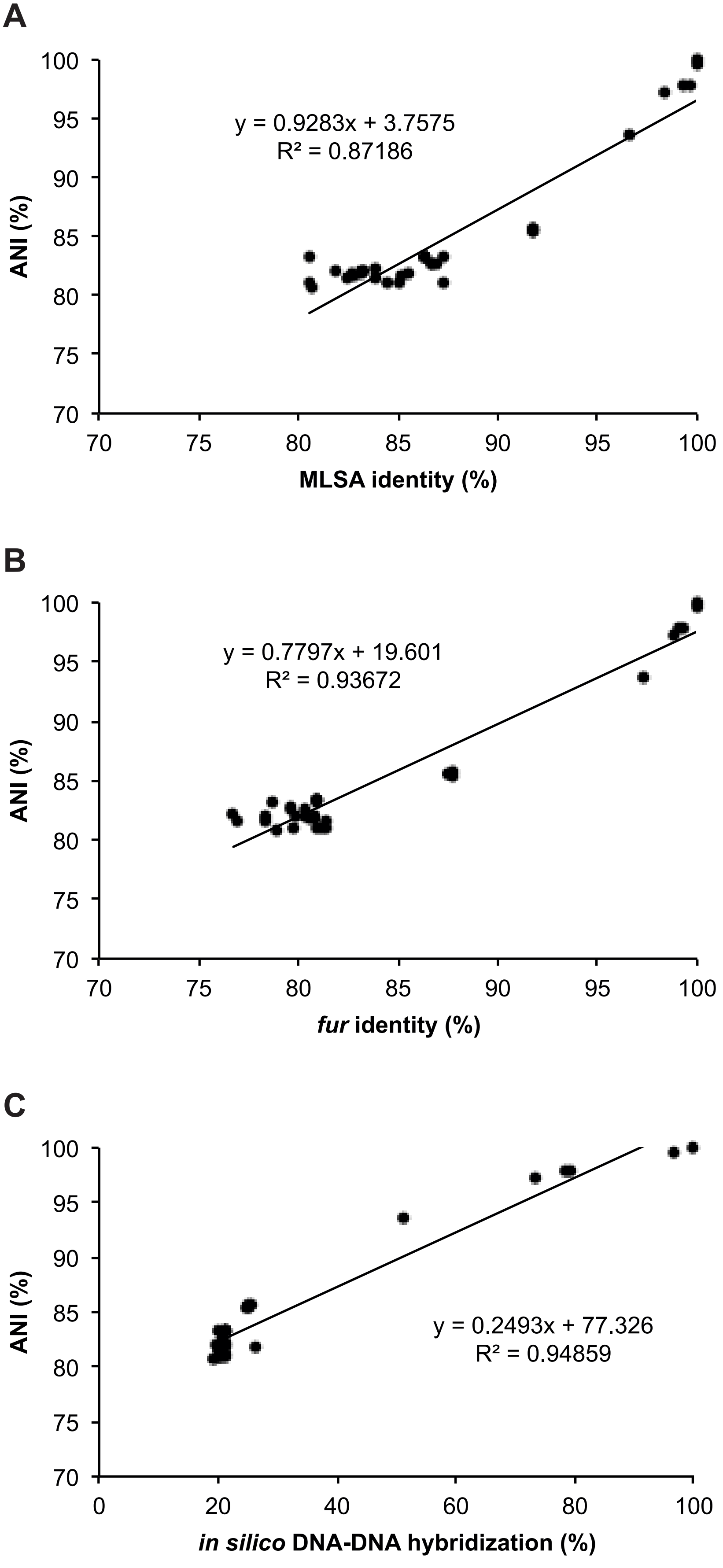

Supplement: Supplementary file 8 [file Image4.PDF]
