# Supplementary material for: Comparative Genomics Reveals High Genomic Diversity in the Genus Photobacterium
Source: Front Microbiol. 2017 Jun 29;8:1204. doi: 10.3389/fmicb.2017.01204 (PMC5489566; doi:10.3389/fmicb.2017.01204)

Figure S5. Different organizations of the *lux-rib* operon in *Photobacterium*.
P. kishitanii, *P. leiognathi* subsp. *mandapamensis* svers.1.1., and *P. phosphoreum* ANT-2200 (a); *P. phospohoreum* ATCC 11040 (b); *P. leiognathi* ATCC 25521, ATCC 33979 and Irivu 4.1 (c).

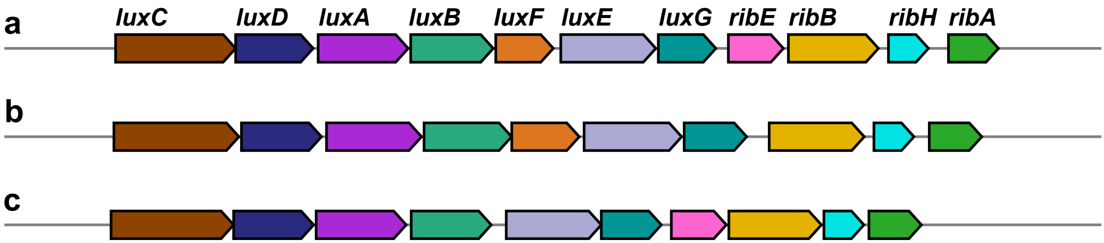

Supplement: Supplementary file 9 [file Image5.PDF]
