# Supplementary material for: Comparative Genomics Reveals High Genomic Diversity in the Genus Photobacterium
Source: Front Microbiol. 2017 Jun 29;8:1204. doi: 10.3389/fmicb.2017.01204 (PMC5489566; doi:10.3389/fmicb.2017.01204)
Supplement: Supplementary file 10 [file Image6.PDF]

Photobacterium profundum SS9

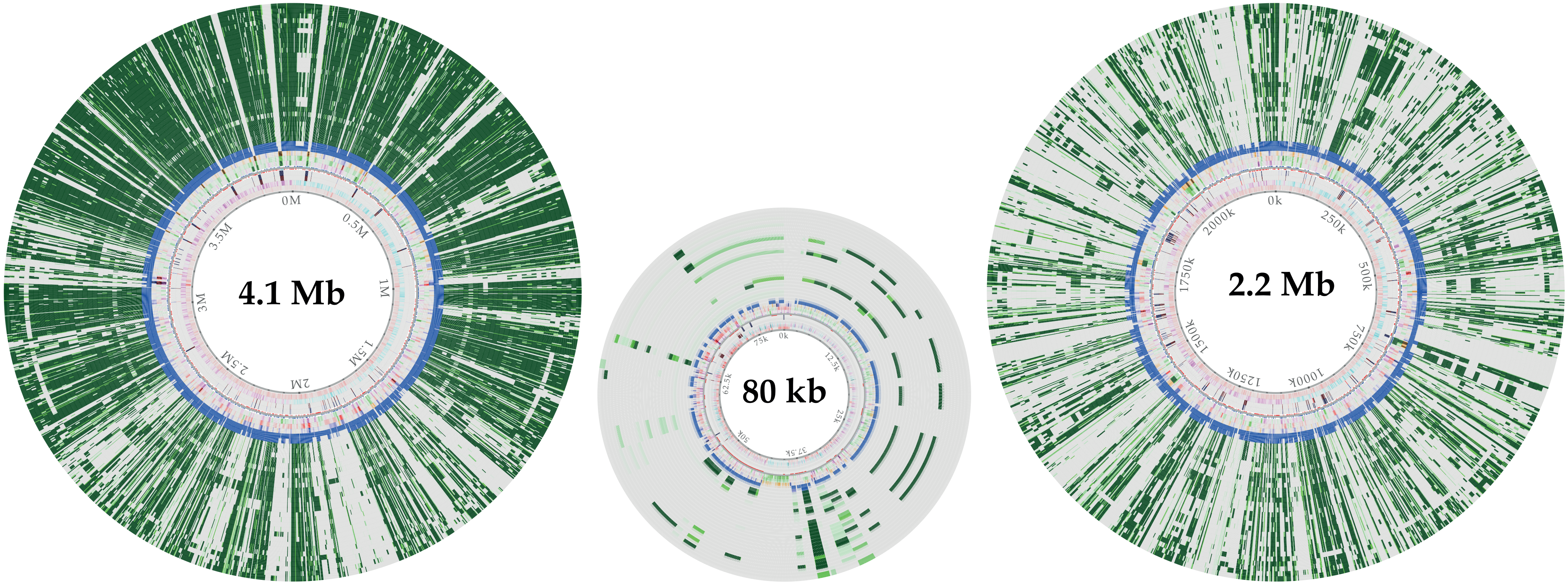

Photobacterium gaetbulicola Gung47

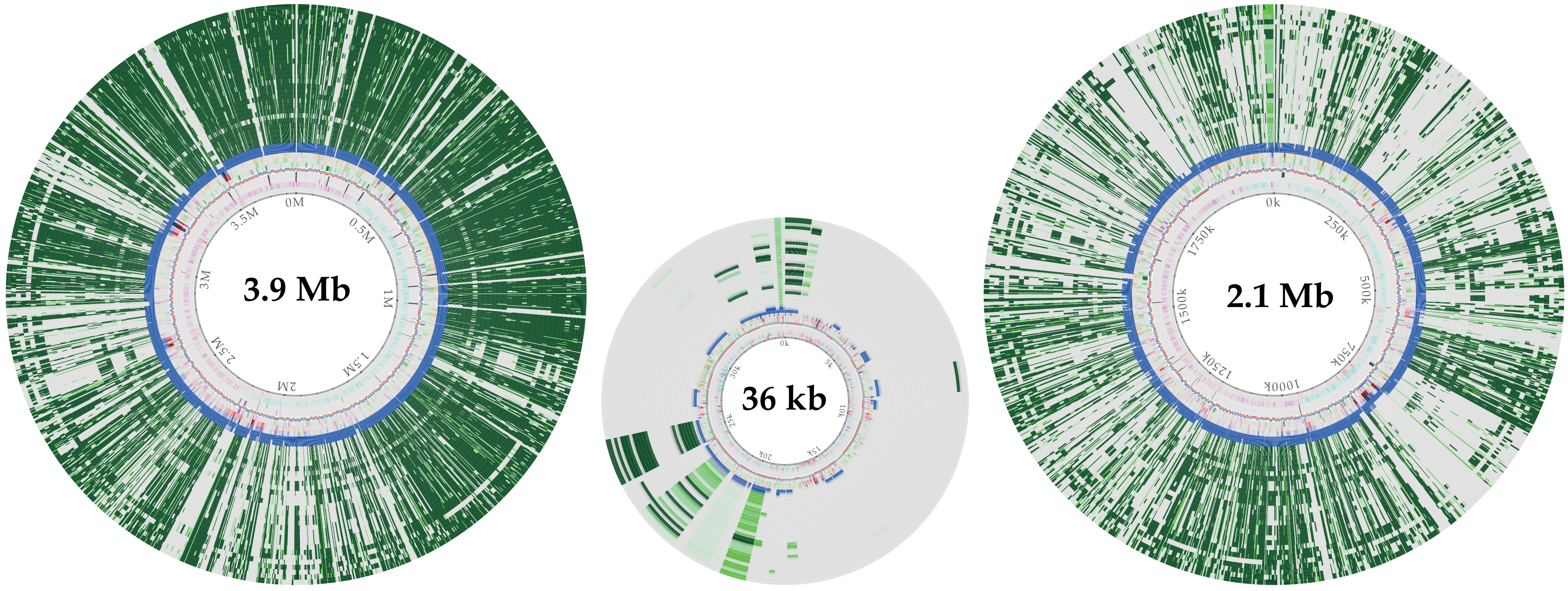

Figure S6. BLAST atlas using the chromosomes and plasmids of *P. profundum* SS9 and *P. gaetbulicola* Gung47 as reference strains. The first blue lane is the reference genome used, followed by other strains of the same species also colored in blue. The remaining species and strains are colored in green and appear in the outter rings of the atlas.
