# Supplementary material for: Comparative Genomics Reveals High Genomic Diversity in the Genus Photobacterium
Source: Front Microbiol. 2017 Jun 29;8:1204. doi: 10.3389/fmicb.2017.01204 (PMC5489566; doi:10.3389/fmicb.2017.01204)

Figure S7. Cluster encoding the proteins responsible for histamine production in *Photobacterium*.

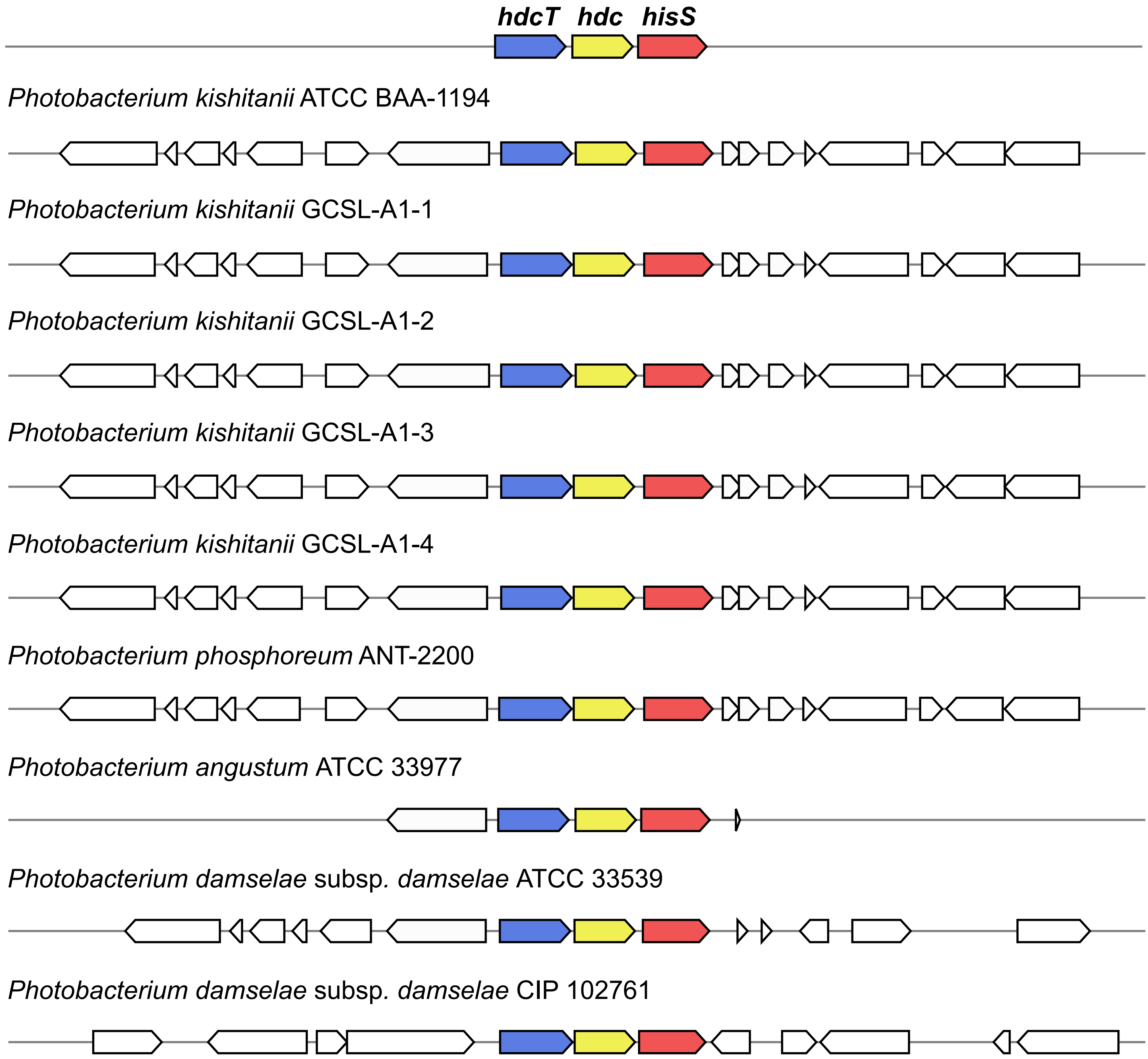

Supplement: Supplementary file 11 [file Image7.PDF]
