# Supplementary material for: Comparative Genomics Reveals High Genomic Diversity in the Genus Photobacterium
Source: Front Microbiol. 2017 Jun 29;8:1204. doi: 10.3389/fmicb.2017.01204 (PMC5489566; doi:10.3389/fmicb.2017.01204)

Figure S8. Power-law regression fitting the pan-genome of *Photobacterium*.
The values were plotted and the equation of correlation is presented.
The R^2 represents the correlation coefficient.

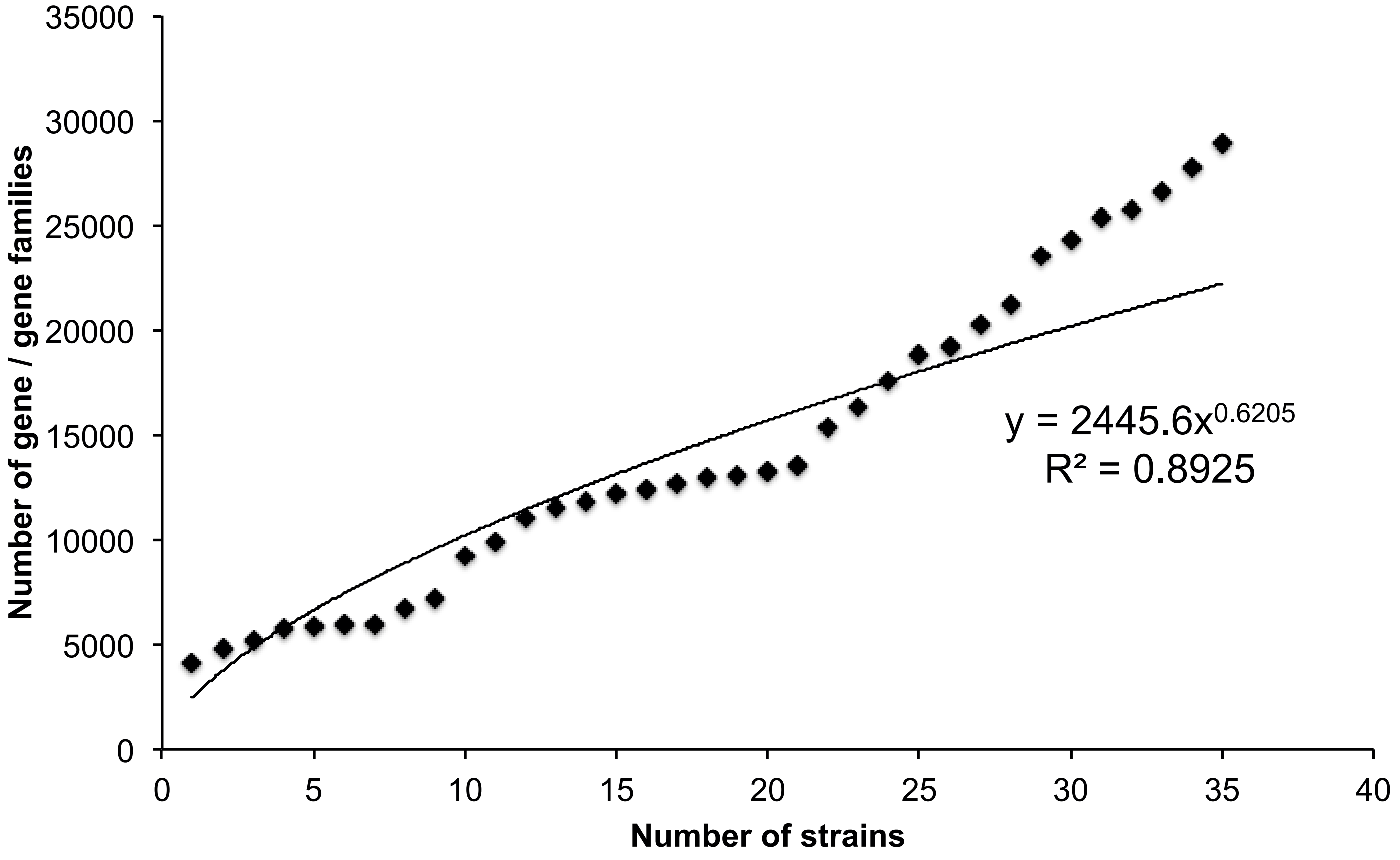

Supplement: Supplementary file 12 [file Image8.PDF]
